# Bioinformatic Analysis of the Wound Peptidome Reveals Potential Biomarkers and Antimicrobial Peptides

**DOI:** 10.3389/fimmu.2020.620707

**Published:** 2021-02-03

**Authors:** Erik Hartman, Karl Wallblom, Mariena J. A. van der Plas, Jitka Petrlova, Jun Cai, Karim Saleh, Sven Kjellström, Artur Schmidtchen

**Affiliations:** ^1^ Division of Dermatology and Venereology, Department of Clinical Sciences, Lund University, Lund, Sweden; ^2^ LEO Foundation Center for Cutaneous Drug Delivery, Department of Pharmacy, University of Copenhagen, Copenhagen, Denmark; ^3^ Dermatology, Skane University Hospital, Lund, Sweden; ^4^ Division of Mass Spectrometry, Department of Clinical Sciences, Lund University, Lund, Sweden; ^5^ Copenhagen Wound Healing Center, Bispebjerg Hospital, Department of Biomedical Sciences, University of Copenhagen, Copenhagen, Denmark

**Keywords:** peptidomics, mass spectrometry, bioinformatics, biomarkers, wound healing, wound infection, antimicrobial peptide, hemoglobin

## Abstract

Wound infection is a common and serious medical condition with an unmet need for improved diagnostic tools. A peptidomic approach, aided by mass spectrometry and bioinformatics, could provide novel means of identifying new peptide biomarkers for wound healing and infection assessment. Wound fluid is suitable for peptidomic analysis since it is both intimately tied to the wound environment and is readily available. In this study we investigate the peptidomes of wound fluids derived from surgical drainages following mastectomy and from wound dressings following facial skin grafting. By applying sorting algorithms and open source third party software to peptidomic label free tandem mass spectrometry data we provide an unbiased general methodology for analyzing and differentiating between peptidomes. We show that the wound fluid peptidomes of patients are highly individualized. However, differences emerge when grouping the patients depending on wound type. Furthermore, the abundance of peptides originating from documented antimicrobial regions of hemoglobin in infected wounds may contribute to an antimicrobial wound environment, as determined by *in silico* analysis. We validate our findings by compiling literature on peptide biomarkers and peptides of physiological significance and cross checking the results against our dataset, demonstrating that well-documented peptides of immunological significance are abundant in infected wounds, and originate from certain distinct regions in proteins such as hemoglobin and fibrinogen. Ultimately, we have demonstrated the power using sorting algorithms and open source software to help yield insights and visualize peptidomic data.

## Introduction

Infected and slow-healing wounds are one of the major costs of the healthcare industry, with some estimates stating that 2–4% of the total healthcare expenditure in Europe is being dedicated to wound care (1, 2). These costs are the result of prolonged hospital stays, more nursing care and dressing changes as well as the prescription of antibiotics and antimicrobial substances (3). Improving diagnostic tools, enabling early prevention of infection, would reap great benefit for the individual patients as well as society. Recent technology advances have led to the development of proteomic approaches enabling the study of the physiology and status of wounds (4–6). Moreover, in order to study the protease dynamics of wounds, comparative degradomics approaches studying the N-terminal proteome have been developed (7, 8).

We have recently developed a peptidomics method for the characterization of endogenous peptides of wound fluids. We compared acute non-infected wound fluids with plasma samples and found significantly higher protein and peptide numbers in wound fluids compared with plasma, which typically were also smaller in size as compared to plasma-derived peptides. We also analyzed wound fluids collected from dressings after facial surgery and demonstrated the utility of peptidomics in wound fluid analysis, showing specific peptide-patterns of various selected proteins, such as those involved in coagulation and complement activation. Together, the work defined a workflow for analysis of peptides derived from human wound fluids, demonstrating a proof of concept that such wound fluids can be used for analysis of subtle qualitative differences in peptide patterns derived from individual patient samples (9). Qualitative analyses of the peptide fragmentation patterns using peptigrams yielded apparent differences between the individual patient samples, suggesting that such datasets could act as potential biomarkers for assessment of infection and inflammation during wound healing. However, it still remained to be investigated whether bioinformatic approaches applied on the *whole* datasets would provide additional information. We particularly focused on establishing algorithms and strategies to define potential biomarkers that could be utilized in future clinical studies. As antimicrobial defense and innate immunity is intimately linked to wound healing another goal of the work was to explore whether there could be alterations in global patterns of antimicrobial peptides (AMP).

Peptides are generated as a result of the interaction between protein substrates and proteases, making peptidomics especially well-suited to study highly proteolytic environments such as wounds. Peptidomic analysis therefore have a potential to complement and extend the existing knowledge gained from proteomics by providing a different perspective on physiological events. From an analytical perspective, the field of peptidomics is particularly suitable for the implementation of bioinformatics, as shown by already existing databases and tools (10). Methods commonly used in proteomics, such as mass spectrometry (MS), naturally translates well into the field of peptidomics. In this study we therefore used an objective bioinformatic approach to investigate datasets generated by tandem mass spectrometry (MS/MS) on the peptidome of acute wound fluids, non-infected wounds and wounds infected with *Staphylococcus aureus*. Our aim was to gain new insight into the physiology and pathophysiology of wounds, as well as to identify contenders for biomarkers. We also aimed to establish and validate a workflow for investigating the peptidome using MS/MS data and simple algorithms in Python, as well as demonstrate how open source software like Deep-AmPEP30 (11), Proteasix (12) and Peptigram (13) may be used to support peptidomic research and help gain novel insights.

Using these approaches, we hereby describe a comprehensive characterization of the wound fluid peptidome from acute surgical wounds of different types. Furthermore, by doing a literature search aided by algorithms we showed that biomarkers and antimicrobial peptides are clustered in specific regions of proteins. Interestingly, an abundance of established AMPs derived from the well-known region in hemoglobin subunit beta (112–147) (HBB) (14–18) as well as peptides derived from LPS-binding regions of hemoglobin were found in infected surgical wounds (19). Additionally, by utilizing large scale antimicrobial prediction by AmPEP, we found that peptides predicted to be antimicrobial were particularly identified in infected surgical wounds, as compared to non-infected and sterile acute wound fluids. Taken together, we demonstrate the power of using an unbiased and simple data driven approach to wound fluid peptidomics and present novel insights regarding wound environment.

## Methods

### Sample Collection and Peptide Extraction

**Figure 1:**
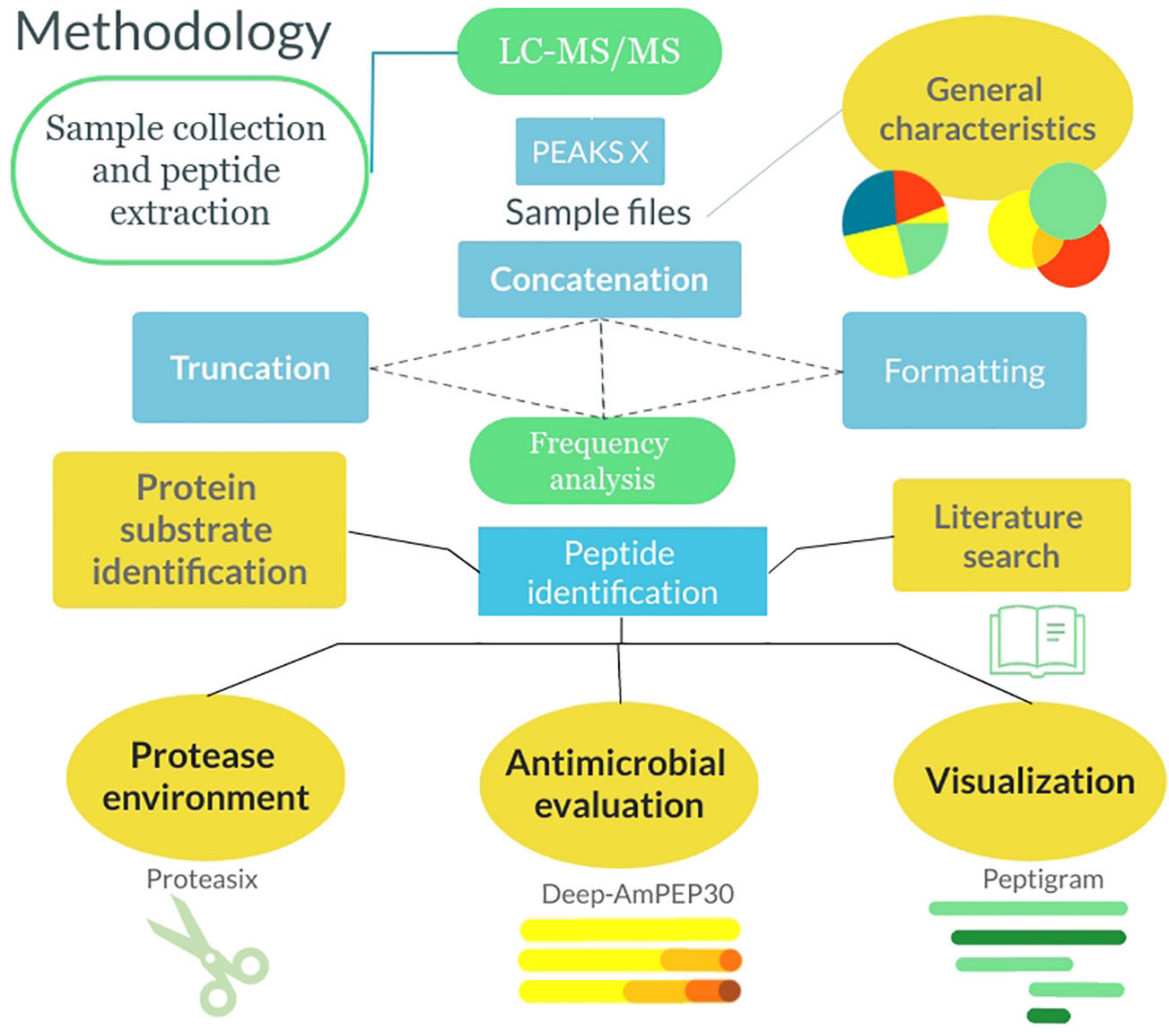
Flowchart describing the methodology from sample collection to data analysis. The samples were collected, and the peptides were extracted. Following this, the samples were analyzed by LC-MS/MS and run through PEAKS X. Thereafter the general characteristics of the peptidomes were extracted and the files were formatted and sorted in Python. Lastly, analysis was done using algorithms in Python as well as utilizing 3^rd^ party software.

The overall workflow is illustrated in **Figure 1**. Plasma was collected from citrated venous blood from healthy donors, sterile acute wound fluids were obtained from surgical drainages after mastectomy, and wound fluids from patients that underwent facial full-thickness skin grafting were extracted from Mepilex^®^ wound dressings (Mölnlycke Health Care AB, Sweden) as previously described (9, 20). The materials were kept frozen at −20°C before use. For peptide extraction, samples were defrosted, and mixed with 8 M urea supplemented with *Rapi*Gest SF (Waters, USA) and incubated for 30 min at RT followed by size exclusion using filters with 30 kDa cut-off (Microcon 30, regenerated cellulose, Millipore, Ireland) as previously described (9). The filtrates containing the peptides were stored at −20°C before analysis by LC-MS/MS.

### LC-MS/MS Analysis

LC-MS/MS experiments were performed using an Orbitrap Fusion Tribrid MS system (Thermo Scientific) as described previously (9). Subsequent analysis was also performed using an HFX Orbitrap MS system (Thermo Scientific) equipped with a Dionnex 3000 Ultimate HPLC (Thermo Fisher). Injected peptides were trapped on an Acclaim PepMap C18 column (3 µm particle size, 75 µm inner diameter x 20 mm length). After the elution the peptides were introduced into the mass spectrometer and analyzed as previously described (9). Briefly the capillary temperature was set at 275°C. Data acquisition was carried out using a top 20 based data-dependent method. MS was conducted in the range of 350–1,350 *m/z* at a resolution of 120,000 FWHM. The filling time was set at a maximum of 100 ms with limitation of 3 x 10^6^ ions. MSMS was acquired with a filling time maximum 300 ms with limitation of 5 x 10^4^ ions, a precursor ion isolation width of 2.0 *m/z* and resolution of 15,000 FWHM. The normalized collision energy was set to 28%. Only multiply charged (2+ to 5+) precursor ions were selected for MS2. The dynamic exclusion list was set to 30 s.

### Application of Sorting Algorithms on Peptidome Data

The data from MS/MS was analyzed in PEAKS X (21) combined with the NCBI Human_20413_20190124 proteomic database, generating a dataset consisting of 11 samples and 3 different file-types, as described below. The retrieved files were analyzed further using algorithms in Jupyterlab using the Python 3.7.5 kernel in the Jupyterlab 2.1.5 IDE. The libraries used for analysis were Pandas 1.0.5, Matplotlib 3.2.2, Numpy 1.18.5 and Seaborn 0.10.1 (requirements and code available at GitHub: https://github.com/ErikHartman/2020-peptidomics). To validate the various preparations of the dressing and acute wound fluids, and to allow for further inter-group semi-quantitative analysis, the absolute quantities of the total spectral count were compared between the different groups. Spectral counting was utilized in order to maximize the hits for peptides of lower abundance (22).

### Characterizing the Peptidomes

Due to the variability in data dependent acquisition the replicate injections of the same sample (n=4) were concatenated before the database search. To reduce the number of false positive identifications, a spectral count value cutoff was applied to the dataset. The cutoff was set to ≥ 4, in line with the reasoning from Lundgren et al. (23). Using the inherent methods of Pandas, the amino acid profile was determined for the different groups. The frequency of a specific letter was multiplied with the spectral count for the respective sequence. The amino acids were then grouped according to side chain properties (24). The complete sequence as well as the P1 and P1’ position was analyzed. The amino acid composition of the complete SWISS-Prot database was included in the dataset as a reference (25). When counting the frequencies of P1 and P1’ amino acids, peptides derived from the N and C-terminals of the origin-proteins were removed from the dataset to only include cleaved sequences. To investigate what protein substrates resulted in the most peptides in each group, the cumulative spectral count of each unique protein was obtained, and the top 10 proteins were plotted in pie charts. Noting that hemoglobin-derived peptides were overly abundant in the infected and non-infected samples, pie charts were made after discarding peptides deriving from hemoglobin subunit alpha, beta, and delta. To detect sequences with outlying spectral count, the spectral count of the sequences in each group was plotted over the number of occurrences across patients, using matplotlib. The retention time of outliers was controlled in all samples to detect false positives. N and C-terminals were set to the 4 proximal amino acids of each terminal in line with the MEROPS database standard (26). The spectral count distribution of unique N and C-terminals were analyzed and plotted with each N and C-terminal as a datapoint. The terminal amino acids were then grouped according to side chain properties (24) and the distribution of grouped N and C-terminals were analyzed and plotted in a similar manner.

The inter and intra-group variance was visualized using Venn diagrams. The 3-way Venn diagrams were visualized using Python, whereas the 5-way Venn diagram had to be visualized using InteractiVenn (27). The intersectional peptides (i.e. the peptides found in all samples within a group) were identified. To identify the peptides differing the most in relative quantity between each group, the difference between the spectral counts from the intersectional peptides in the infected and non-infected samples respectively were calculated. The 50 peptides yielding the largest difference were formatted and run through the Proteasix open source tool for protease prediction. Proteasix is a peptide-centric tool based on a cleavage site database, built through CutDB, Uniprot and literature, which utilizes knowledge of protease preference from e.g. the ENZYME database to predict the responsible protease for a peptide from a specific protein substrate (12).

### Literature Search

To validate our findings an extensive database search was conducted using PubMed (28), Elsevier’s ScienceDirect (29) and LUBsearch (30), using relevant search terms such as “Peptide”, “Biomarkers”, “Peptidome” and “Antimicrobial”. The peptide sequences presented in the articles were saved if the article was deemed of sufficient quality and relevance. Using a Python script, the gathered data was cross-checked against matching sequences in our datasets. In **Supplementary Table 1** we display all input data for the script and all matching sequences.

### Antimicrobial Peptide Prediction Using Deep-AmPEP30

The antimicrobial propensity was investigated in the different groups. The unique peptides were run through the novel Deep-AmPEP30 algorithm, a classification model using reduced amino acid composition and convolutional neural networks to predict short AMPs (11). Peptides with a Deep-AmPEP30 score of <0.7 were truncated from the dataset. Thereafter, the spectral count for each sample was multiplied with the respective Deep-AmPEP30 score to retrieve the antimicrobial score. The scores for the various samples were visualized in a heatmap using Seaborn. To investigate what protein substrates resulted in AMPs, the peptides with a Deep-AmPEP30 score ≥ 0.7 were grouped on their respective protein and a pie chart was created from the 10 most common proteins.

### Visualization Using Peptigram

Three proteins, fibrinogen A, hemoglobin subunit beta and hemoglobin subunit alpha, were identified as of interest and were further investigated and visualized using Peptigram (13) **(** **Supplementary Data Sheet 1** **)**. To produce the Peptigrams, peptides derived from these proteins were singled out and formatted to be inserted into the Peptigram website (http://bioware.ucd.ie/peptigram/). Peptigram is a free-to-use web-based software developed to visualize differences between peptidomic samples using peptide alignment maps and profiles.

### Statistical Methods

The paired Student t-test was applied using GraphPad when deemed appropriate (31). The degrees of freedom were set to N-1 for all standard deviations and were calculated using Pandas and Numpy.

### Dataset

The dataset retrieved from MS/MS when run through PEAKS X combined with the NCBI Human_20413_20190124 proteomic database resulted in 3 types of xlsx-files per sample: peptide-files, protein-peptide-files and protein-files (available at https://github.com/ErikHartman/2020-peptidomics). Each filetype contained some unique information regarding sequence and protein-distribution and was utilized when deemed appropriate. The raw datafiles have been submitted to ProteomeExchange (PXD023244, http://www.proteomexchange.org/).

## Results

### General Characteristics of the Peptidomes

Our data consists of 2 types of clinical samples, sterile acute wound fluid (n=5) and fluid extracted from non-sterile wound dressings (n = 6). The samples constituting the wound dressing group differed, as half (n = 3) of the wounds were healthy whereas half infected by *S. aureus*, confirmed both clinically and by wound culture. The total spectral count between the groups did not vary significantly, validating further inter-group semi-quantitative analysis **(** **Supplementary Figure 1** **)**.

**Figure 2:**
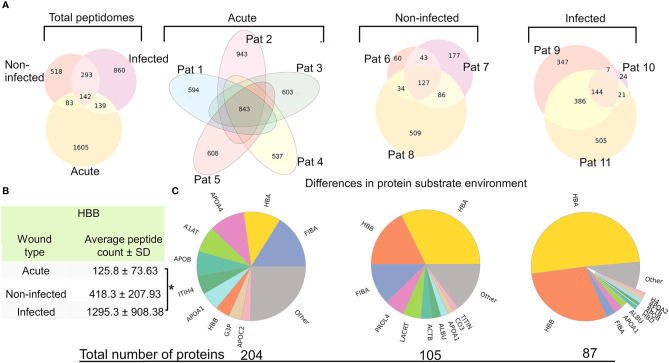
Large intra and inter group peptide variance and differences in protein substrate distribution **(A)** Venn-diagrams depicting the intra and inter group variance of peptide sequences, after employing a spectral count cut off. The leftmost diagram shows the intergroup variance, whereas the other depict the intragroup peptidomic variances. **(B)** Table showing the average amount of peptides derived from hemoglobin subunit beta (HBB) in the groups, showing significantly more peptides derived from HBB in the infected group as compared to the acute wound fluid group (p=0.0231) **(C)** Pie charts of the protein substrate environment in the different groups made by counting the total spectral count of all peptides associated to its parent protein. The numbers below the pie charts are the total number of proteins that have one or more peptides.

Due to the varying wound environments between the groups, we hypothesized that there could be differences in the general characteristics, such as average peptide length and mass, of the peptidomes. As seen in **Supplementary Table 2** there was neither any significant difference regarding the average mass (1,319–1,439 kDa) nor the average length (12.06–13.41 amino acids). The same applied to the average number of peptides in each group without reducing signal noise (spectral count cutoff ≥ 4) (acute: 3,331 ± 686, non-infected: 4,095 ± 873, infected: 3,815 ± 2,083). When employing the spectral count cut off, the number of unique peptides found was reduced dramatically (acute: 812 ± 143, non-infected: 1,006 ± 250, infected: 1,434 ± 455), showing that many peptides are false positives and/or of low quantity. Acute wound fluid contained the least number of peptides per sample but had the largest fraction of peptides above the spectral count cutoff (28.3%), whereas the non-infected samples had the most peptides per sample but the smallest fraction of peptides above the spectral count cutoff (15.0%). Plotting the spectral count for all samples revealed some outlying sequences with consistent retention time, which are presented in **Supplementary Figure 2**. **Figure 2A** shows the inter and intra group variance of found peptides when regarding the intersectional peptides as compared to the total unique peptides in a given group or between groups (acute: 124 (6.1%), non-infected: 127 (8,1%), infected: 144 (10.0%), between groups: 142 (3.9%)). Notably, the peptidome of patient 10 is substantially smaller and contains very few unique peptides as compared to patient 9 and 11.

### Differences in Proteomic Diversity

Identifying which protein substrates give rise to peptides in the different wound environments may reveal interesting aspects of the wound’s proteome and proteolytic dynamics. Therefore, a proteomics perspective was applied to the data by summing up the spectral count of peptides originating from the same protein. The relative abundance of proteins originating in peptides was plotted in a pie chart (**Figure 2B** **).** The groups varied regarding protein diversity, and the group difference regarding the proportion constituting hemoglobin derived peptides is especially distinct, as they were found predominantly in the infected samples and barely at all in acute wound fluids. To reduce the influence of hemoglobin on proteomic diversity, pie charts were created after discarding peptides associated with hemoglobin **(** **Supplementary Figure 3** **)**. The results revealed the presence of histone 2 and 4 derived peptides in the infected samples.

### Amino Acid Profiles of the Peptidomes

**Figure 3:**
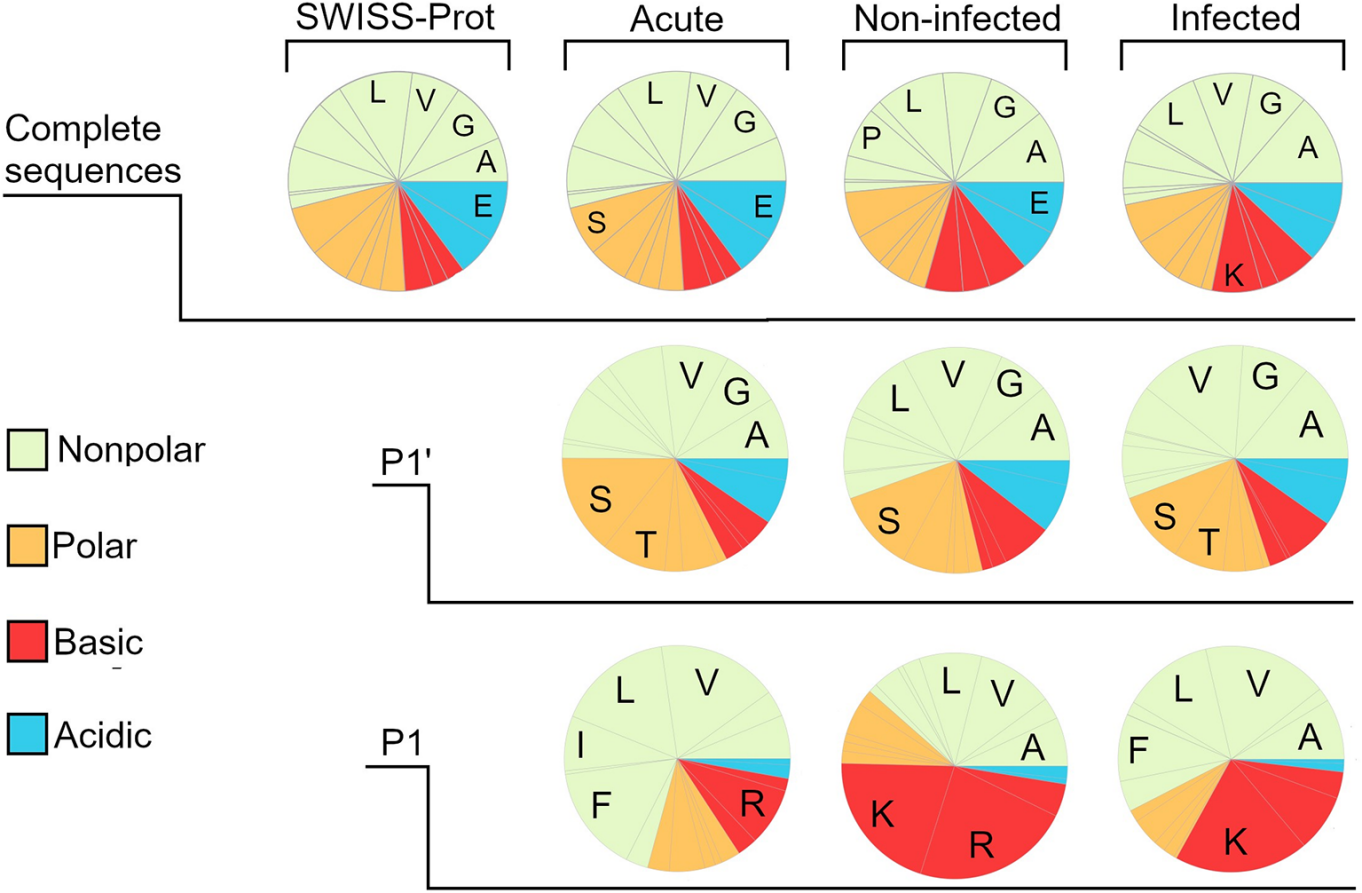
Amino acid composition of the different wound fluid peptidomes. Pie charts created by frequency analysis of amino acids in the complete sequence, weighting the results by the relative quantity (spectral count) of the peptides. Similarly done for the last position (P1’) and first position (P1) but excluding those peptide sequences corresponding to the protein substrate inherent N- and C-terminal, since they are not generated by proteases. For reference, a pie chart was made by using data on the whole SWISS-prot database, showing a distinct similarity regarding the amino acid distribution in whole sequences. The most noticeable difference between the different groups can be seen when looking at the P1 position, showing a much larger proportion of acidic amino acids in both the infected and non-infected. The five most prominent amino acids are denoted by one-letter abbreviations.

To investigate whether the distribution of amino acids differed between the groups, the sequences were truncated and a frequency analysis of the amino acids in the first (P1’) and last (P1) position as well as in the complete sequence was conducted (**Figure 3**, **Supplementary Table 3** **)**. Inherent N and C-terminals of protein substrates were not included in the analysis. A reference for the amino acid distribution of complete protein-sequences was created by using data from SWISS-Prot. As can be seen in the amino acid-profile, the complete sequences were nearly identical to the reference. However, differences were seen when comparing the groups, as wound fluids derived from dressings contained a larger proportion of acidic residues in the P1 position. In **Supplementary Figure 4**, data from *in vitro* experiments by Saravanan et al. (32) on *S. aureus* aureolysin degradation of thrombin are presented. The data showed that the wound fluids extracted from bacteria containing wounds showed similar profiles to the *in vitro* digested samples in the P1 position.

### Prevalence of Specific N and C-Terminals

**Figure 4:**
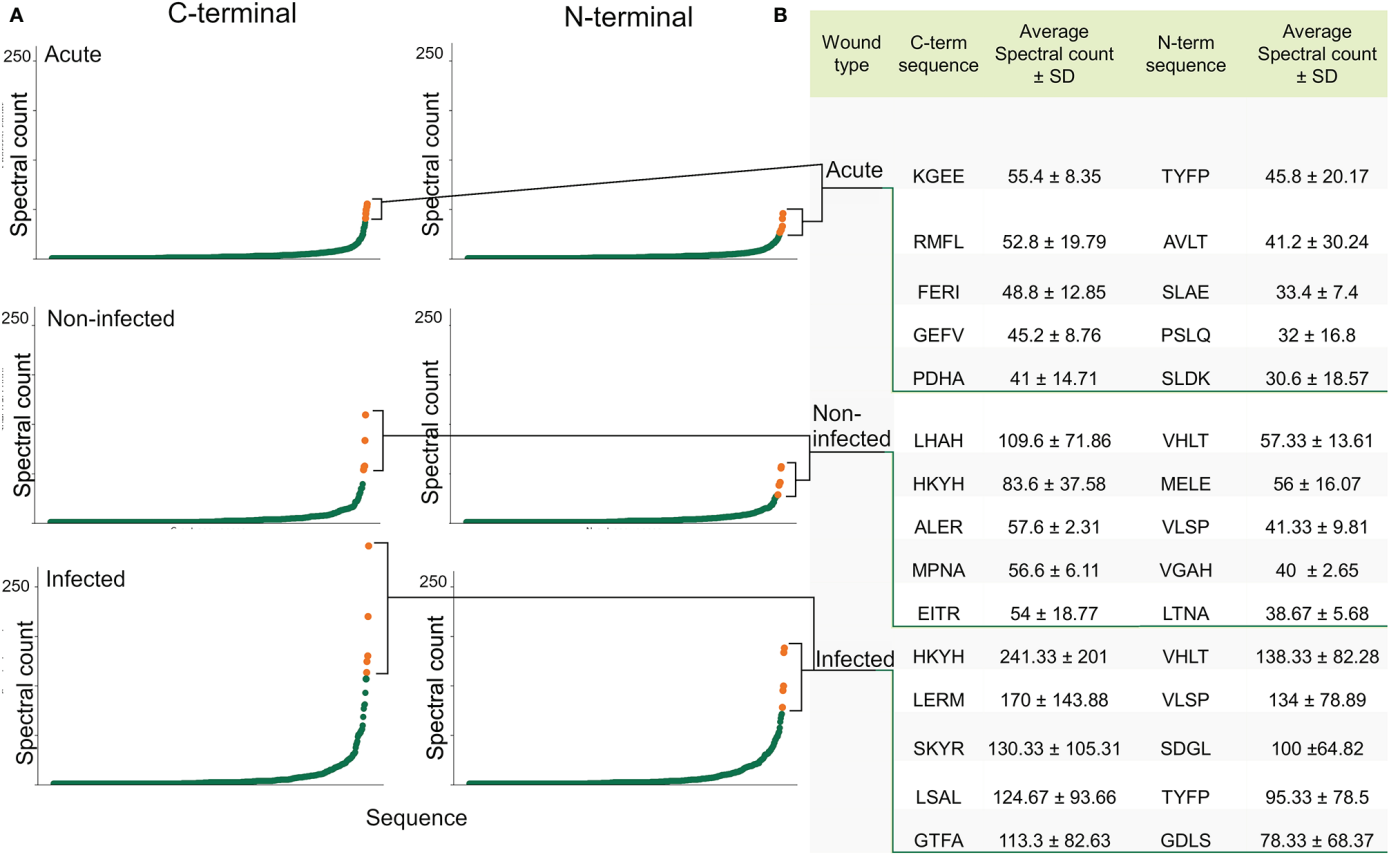
Identification of the most abundant N- and C-terminals. **(A)** Scatter plots visualizing the distribution of the present N-terminals (the first 4 amino acids) and C-terminals (the 4 last amino acids) by plotting the aggregated spectral count of the unique terminal sequences. A difference in curvature in the different groups can be seen. **(B)** Table showing the sequence and average spectral count of the most abundant terminals, which are highlighted in the scatter plots.

The N- and C-terminal of peptides are especially interesting since most peptides are generated post-translationally by the interaction between proteases and substrate proteins. To investigate the distribution of different N- and C-terminals between the groups, a frequency analysis of terminal segments, looking only at the first and last 4 amino acids, was conducted, aggregating the spectral count of all peptides with the corresponding unique terminal sequence. Sequences derived from the protein substrates inherent N and C-terminal were not discarded from the dataset, as they are also of interest when looking for biomarkers. **Figure 4A** was created by plotting the aggregated spectral count of the unique terminal sequences. The steep curvature presented in all graphs indicated a preference for certain sequences. When comparing the groups, differences in the shape of the curvature was noticed, with an especially steep curve in the infected group. The sequences constituting the tip of the curve varied between the groups, although there was an overlap. At the C-terminal, sequences like HKYH, LERM and SKYR were especially prevalent in the infected group (241 ± 201, 170 ± 144, 130 ± 105), while sequences like KGEE, RMFL and FERI are most prevalent in acute wound fluid (55 ± 8, 43 ± 20, 48 ± 13) (**Figure 4B**). The sequences were then grouped according to their side-chain polarity and acidity, yielding similar results **(** **Supplementary Figure 5** **)**.

### Protease Prediction Using Proteasix

The differing peptidomes may be due to either varying protein substrate concentrations or protease environments. To investigate the possible differences in the protease environment, the protease prediction tool Proteasix was used on the 50 most distinct peptides in the infected and non-infected samples. Proteasix predicts the protease responsible for generating a specific peptide from a specific protein using an extensive cut-site database, resulting in a generated list of proteases likely to be responsible for the peptidome (12). The top 5 proteases responsible for generating the 50 most distinct peptides in the samples were: Infected: PGA3, ELANE, Ctsl, Capn1 and Capn2. Non-infected: Capn2, Capn1, MMP7, PGA3, and Ctss respectively. Full list is found in **Supplementary Table 4**.

### Peptides Validated Through Previous Studies

Due to our unbiased approach and limited dataset, an extensive literature search was conducted to validate and put our results into perspective. First, a basal search was made utilizing google, searching on the peptide sequences primarily found in the infected samples. Interestingly, this showed that many of the peptides originated from the hemoglobin subunit beta C-terminal (region 112-147 VCVLAHHFGKEFTPPVQAAYQKVVAGVANALAHKYH) which has been documented as AMPs (14–18). To complement these results, a more structured literature search was made by searching after matches on peptides found in previous studies in our data, utilizing a Python script **(** **Supplementary Table 1** **)**. The results from this search can be seen in **Table 1**, showing that peptides previously identified as biomarkers for various inflammatory diseases indeed are present in the wound samples to varying degrees.

**Table 1:** Peptides from hemoglobin subunit beta and alpha, as well as fibrinogen alpha validated through previous studies.

Region	Previous found significance	Total spectral count WF	Total spectral count NINF	Total spectral count INF
**HBA (129–142) FLASVSTVLTSKYR** (1) FLASVSTVLTSK (2) KFLASVSTVL (3) SVSTVLTSKYR	(1) Upregulated in hypertrophic scar tissue (33) (2) Biomarker cutaneous lupus erythematosus (34) (3) LPS-binding (19), upregulated in hypertrophic scar tissue (33)	**34** (1) 0 (2) 0 (3) 0	**118** (1) 13 (2) 0 (3) 7	**305** (1) 0 (2) 0 (3) 45
**HBA (111-129)** (1) AAHLPAEFTPAVHASLDKF (2) AHLPAEFTPAVHA	**(1) Found in cervicovaginal fluid and shown to potentiate smooth muscle contractions (** 35 **)** (2) LPS-binding (19)	**59** (1) 0 (2)0	**66** (1) 6 (2)5	**162** (1) 21 (2)13
**HBA (1-29) MVLSPADKTNVKAAWGKVGA HAGEYGAEA** (1) VLSPADKTNVKAAWGK (2) VLSPADKTNVK (3) TNVKAAWGK	**Generated by candidal aspartactic peptidases and has bactericidal effect (** 36 **)** (1) Upregulated in hypertrophic scar tissue (33) (2) Urinary biomarker acute severe pancreatitis (37), LPS-binding (19) (3) Upregulated in hypertrophic scar tissue (33)	**29** (1) 0 (2) 0 (3) 0	**211** (1) 4 (2) 33 (3) 0	**844** (1) 5 (2) 52 (3) 8
**HBA (32-93) RMFLSFPTT KTYFPHFDLS HGSAQVKGHG KKVADALTNA VAHVDDMPNA LSALSDLHAH KLR** **(1) SFPTTKTYFPHFDLSHGSAQVK** **(2) TYFPHFDLSHGSAQVKGHGKK** **(3) TYFPHFDLSHGSAQVK**	**Found to have a role to protect against infection in the vagina (** 38 **) Effective against *E. coli*, *Strep. faecalis, Staph. aureus* and *Candida* (** 17 **)** (1) Antibacterial peptide in menstrual blood (14) (2) Upregulated in hypertrophic scar tissue (33) (3) Urinary biomarker acute severe pancreatitis (37).	**575** (1) 0 (2) 0 (3) 16	**613** (1) 0 (2) 0 (3) 22	**1770** (1) 8 (2) 5 (3) 18
**HBB (1-21) MVHLTPEEKSAVTALWGKVNV** **(1) VHLTPEEKSAVTA** **(2) VHLTPEEKSA**	**Generated by candidal aspartactic peptidases and has bactericial effect (** 36 **)** (1) Upregulated in hypertrophic scar tissue (33) (2) LPS-binding (19)	**90** (1) 19 (2) 0	**242** (1) 21 (2) 0	**533** (1) 14 (2) 0
**HBB (112-146) LVCVLAHHFGKEFTPPVQAAYQKVVAGVANALAHKYH** **(1) AGVANALAHKYH** **(2) AHHFGKEFTPPVQAAYQKVVAGVANALAHKYH** **(3) EFTPPVQAAYQKVVAGVANALAHKYH** **(4) NALAHKYH** **(5) VVAGVANALAHKYH** **(6) VAGVANALAHKYH**	**Shown to be antibacterial against both gram-positives and gram-negatives. Antiviral and antifungal properties (** 14, 17, 18 **). Found to play a role in the defense against bacteria in the vagina (** 15 **).** (1) Urinary biomarker Psoriatic arthritis (39) (2) Antibacterial peptide in menstrual blood (14) (3) Urinary biomarker renal cell carcinoma (40) (4) LPS-binding (19) (5) Urinary biomarker acute severe pancreatitis (37), transitional cell carcinoma (41) Upregulated in hypertrophic scar tissue (33) (6)Upregulated in hypertrophic scar tissue (33)	**39** (1) 15 (2) 0 (3) 0 (4) 0 (5) 4 (6) 11	**233** (1) 41 (2) 0 (3) 0 (4) 28 (5) 38 (6) 30	**524** (1) 109 (2) 5 (3) 4 (4) 57 (5) 26 (6) 53
**FIBA (603-624) SYKMADEAGSEADHEGTHSTKR** (1) DEAGSEADHEGTHSTK (2) SYKMADEAGSEADHEGTHST (3) KMADEAGSEADHEGTHST (4) DEAGSEADHEGTHSTKR (5) AGSEADHEGTHSTKRG	(1) Urinary biomarker in Rheumatoid arthritis (39), renal cell carcinoma (40) (2) Serum biomarker preeclampsia (42) (3) Serum biomarker Chrohn’s disease (43) (4) Urinary biomarker in Infantile necrotizing enterocolitis (44) (5) Urinary biomarker Psoriatic arthritis (39)	**0** (1) 0 (2) 0 (3) 0 (4) 0 (5) 0	**70** (1) 5 (2) 0 (3) 0 (4)12 (5) 0	**19** (1) 4 (2) 0 (3) 0 (4) 0 (5) 0
**FIBA (20-35) (Fibrinopeptide A) ADSGEGDFLAEGGGVR** **(1) DSGEGDFLAEGGGV** **(2) ADSGEGDFLAEGGGV** **(3) DSGEGDFLAEGGGVR** **(4) EGDFLAEGGGVR** **(5) FLAEGGGVR**	**Synovial biomarker for Psoriatic arthritis (** 45 **)** (1) Serum biomarker preeclampsia (42), Synovial fluid biomarker for Inflammatory arthritis (45) (2) Serum biomarker preeclampsia (42) (3) Serum biomarker renal cell carcinoma (46), non-small cell lung carcinoma (47), Crohn’s disease (43) (4) Serum biomarker Alzheimer’s disease (48), renal cell carcinoma (46), Crohn’s disease (43) (5) Synovial fluid biomarker for Inflammatory arthritis (45)	**189** (1) 20 (2) 0 (3) 43 (4) 23 (5) 8	**107** (1) 0 (2) 0 (3) 26 (4) 16 (5) 0	**43** (1) 0 (2) 0 (3) 14 (4) 9 (5) 0

An extensive literature search was made using pubmed, elsevier and lubsearch. Documented peptides were extracted and matched with sequences in our dataset, resulting in several matches. Several regions of proteins (mainly Hemoglobin subunit alpha and beta and fibrinogen alpha) yielded peptides associated with antimicrobial properties and biomarker potential. These are presented in the table together with the sum of the spectral count of all sequences confined within the region (in bold), and the spectral count of the exact peptides. Sequences without any exact match in our dataset, but with close similarity to many other sequences are also included.

Furthermore, peptides recognized as biomarkers tend to be clustered around certain regions, as seen in **Table 1**. As mentioned, the hemoglobin subunit beta region (112–147) stands out, having been thoroughly described for its various antimicrobial properties both *in vitro* and *in vivo*, but also as a biomarker for several inflammatory conditions such as hypertrophic scar formation (33), acute severe pancreatitis (37) and transitional cell carcinoma (41). In order to estimate the presence of the various peptides and regions in the different groups, spectral counting was performed. This showed that the quantity of the investigated regions differed between groups, and that documented regions from e.g. hemoglobin was mostly present in infected wound fluids.

### Antimicrobial Peptides of Peptidomes

**Figure 5:**
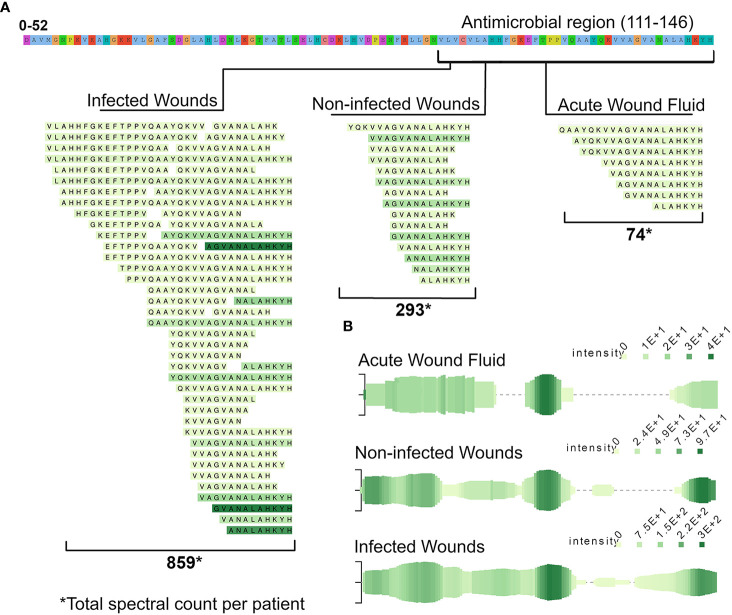
Presence of peptides derived from the antimicrobial C-terminal (112–147) of hemoglobin subunit beta across the different wound fluids. **(A)** To elucidate the difference in peptide expression of the region, a peptide alignment map was made, depicting the found peptides, aligned to the parent sequence of hemoglobin. The color intensity of the peptides reflects the average spectral count in the respective groups, ranging between 1–32. **(B)** Peptide profiles of the whole HBB - protein. Each green line along the x-axis represents an amino acid (AA) in the HBB sequence that is found in a peptide. The vertical size of the line is proportional to the number of peptides containing that AA while the color intensity is proportional to the summed.

As the literature search showed, many peptides detected in our datasets were found to possess antimicrobial properties, such as peptides derived from the abovementioned region in hemoglobin subunit beta. Furthermore, the quantity of peptides derived from this region differed between the groups. To elucidate this further, a peptide alignment map was made of the region using the open source program Peptigram **(** **Figure 5A** **)**. The figure showed that more peptides were generated from this region in the infected samples than in the non-infected samples, and to a much lesser degree in the acute wound fluid group. Furthermore, the peptide profile showed a larger overall abundance and coverage of peptides from hemoglobin subunit Beta in the infected samples **(** **Figure 5B** **)**.

**Figure 6:**
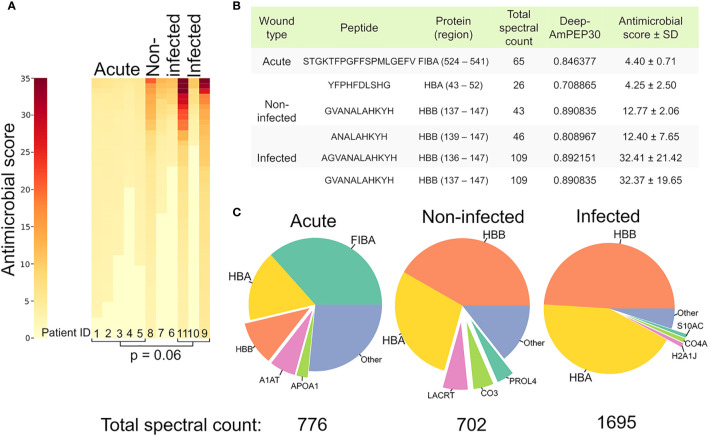
Increased antimicrobial activity in infected samples. **(A)** Heatmap depicting the predicted antimicrobial environment in the different samples. Heat map color is determined by the antimicrobial score, which was calculated by multiplying the retrieved prediction score from Deep-AmPEP30 with the respective spectral count for each sequence. A cut-off value (0.7) was applied on the Deep-AmPEP30 prediction score. **(B)** Table highlighting the peptides with the highest antimicrobial score in each group. **(C)** Pie charts showing the proportions of parent proteins generating the antimicrobial peptides.

Since many of the peptides found in large quantities have not been investigated for antimicrobial properties, *in silico* predictions of antimicrobial activity were made on our complete dataset using a bioinformatic tool available for short peptides (< 30 amino acids), Deep AmPEP-30. This yielded a prediction score of being antimicrobial between 0-1 for each peptide based on its amino acid-sequence **(** **Supplementary Table 5** **)**. In order to only include peptides with a high probability of being antimicrobial, all peptides with a prediction score below 0.7 were discarded. The prediction score was then multiplied with the associated spectral count, yielding an antimicrobial score. The antimicrobial score is an estimation of the peptide’s contribution to the antimicrobial environment. The antimicrobial environment of the different samples was visualized in a heatmap (**Figure 6A**) showing distinctively different antimicrobial environments in the different samples.

The peptides with the highest antimicrobial score in the non-infected and infected samples originate from the antimicrobial region 112-147 in hemoglobin subunit Beta, while they originate from G3P, FIBA and APOA4 in acute wound fluid (**Figure 6B**). To visualize which proteins served as substrates for the AMPs in the different groups, pie charts were made (**Figure 6C**) by summing the total spectral count of all AMPs from each substrate protein above the prediction score cutoff value. As can be seen in **Figure 6C**, the hemoglobin derived AMPs were more prevalent in the wound dressing group as compared to the acute wound fluid group. The total sum of the spectral count of the peptides above the prediction score cutoff value were [acute: 776 (n=5), non-infected: 702 (n=3) infected:1695 (n=3)].

## Discussion

Peptidomics is demonstrably well suited for the implementation of bioinformatics and big data analysis (10). Recognizing patterns and identifying key peptides in different environments will result in a better understanding of conditions like inflammation and infection, as well as protease and protein dynamics. Furthermore, it will enhance our understanding of processes related to wound-healing, which may prove pivotal for future advances in wound care (49). This study demonstrated the efficacy and potential impact of simple sorting algorithms on peptidomic data, and how open source software may be implemented in research. Our datasets were generated through data dependent acquisition, but it may very well be implemented on other types of quantifiable data with a similar structure, although different methods of data-gathering may result in different findings as they often favor certain peptide-characteristics, and inter-methodology comparisons may therefore not be viable.

The field of peptidomics is not yet established to the same extent as seen for proteomics, although contemporary research shows that peptides play a substantial role in many physiological and immunological processes (50–53). To our knowledge, the unaltered wound fluid peptidome has not been thoroughly described, leaving room for our data to complement and extend existing knowledge (54, 55). From an analytical standpoint, previous studies have shown that spectral counting and intensity-based quantification methods both have their merits in peptidomic approaches (56). Although using intensity is accurate and preferred when determining the ratio between identical peptides in quantitative analyses, spectral counting combined with filtering can be applied for inter-peptide approximate quantitative measurements, yielding specificity and sensitivity while reducing the number of false positives. The hit ratio for peptides of lower abundance may thus be improved using spectral counting, which is of importance from a discovery and biomarker perspective, as sequences discarded from the dataset due to intensity cutoffs are kept when utilizing spectral counting, thus motivating its use in this study. When looking at the general characteristics of the whole peptidomes, such as average mass, peptide length and the total number of unique peptides, insignificant differences between groups and samples were seen **(** **Supplementary Table 2** **)**. However, the infected group did contain one outlying sample, with a considerably smaller peptidome than the others. Additionally, there were little differences regarding the total spectral count between groups **(** **Supplementary Figure 1** **)**, validating the sample preparation and allowing for semi-quantitative comparisons between the groups.

Investigating the unique peptides making up the peptidomes demonstrated a high variance in composition, between acute wound fluids from mastectomy drainages and wound fluids extracted from wound dressings of infected and non-infected surgical wounds (**Figure 1A**). Furthermore, the protein substrate environment varied between groups, as the acute wound fluid samples contained a more varied environment as compared to the wound dressings samples which was dominated by hemoglobin, especially in the infected subgroup (**Figures 1B, C**). We also show that there is a striking similarity between the amino acid distribution of the peptidomes and that of the Swiss-Prot database, validating our dataset and the connection between peptidome and proteome.

As protease specificity is foremost determined by the amino acid residues closest to the cleavage sites (P1, P1’) (57), we hypothesized that the distribution of these amino acids could be influenced by differences in the respective protease environments. It was therefore interesting that a difference was indeed observed in the amino acid distribution at the P1 position between the acute wound fluid and wound dressing group. Notably, the preference for acidic residues in the last position in the wound dressing group was similar to the results obtained by Saravanan et al. (32), which used aureolysin in order to fragment thrombin *in vitro*. Taken together, these results serve as a proof of principle that different protease environments can indeed generate detectable differences in global cleavage sites **(** **Supplementary Figure 5** **)**. It is notable that *S. aureus* (9 *)* was only found in the infected wound dressings but not in the acute sterile wound fluids since it was extracted from a sterile environment, providing a possible explanation for the observed differences in cleavage sites and linking the findings from the present study with previous vitro results on aureolysin digested fragments. The samples were searched against a *S. aureus* database, but no significant proteins were found.

The differences seen in the peptidomes are most likely due to a combination of changes in substrate abundancy and protease expression, as peptides are generated in the interaction between these two entities (55). Changes in this dynamic will lead to different peptidomes and might therefore reflect changes in important physiological processes (54, 58). A relevant physiological process exemplifying this is an increase in inflammation due to infection. This will initiate neutrophil activation, introducing potent host-derived proteases such as neutrophil elastase (ELANE), cathepsin G and proteinase 3 (59, 60), altering the protease environment. To investigate whether there was a difference in protease activity between the groups, *in silico* prediction using the open source program Proteasix was conducted. The prediction by Proteasix resulted in mostly the same proteases for both groups with proteases such as PGA3, Capn2, Capn1, Ctsl, MMP7, MMP9, Mep1a and ELANE, being most prevalent **(** **Supplementary Table 4**). These are predominantly proteases known to be associated with inflammation (61–65). Notably, proteases and their inhibitors contribute to the balance between extracellular matrix degradation and deposition, creating an equilibrium that is essential for the timely and coordinated healing of cutaneous wounds. Increased levels of proteolytic enzymes are present in infected wounds, and neutrophil elastase is one major enzyme released from neutrophils invading the infected wound areas (66). It was therefore of interest that ELANE was particularly up regulated in infected samples, serving as a possible indicator of wound infection (67). PGA3 (pepsin) activity was also detected, indicating actions of aspartic peptidases, a widely distributed proteolytic enzyme family (68). Although pepsin per se is only found in the stomach, there could be related endopeptidases present in infected wounds. It should be noted however, that the large amount of hits for several different proteases suggest a low specificity in the Proteasix tool which could not discriminate between our datasets, making it difficult to obtain conclusive results for other proteases.

Inferring conclusions about the protease environment from peptidomic data is made difficult by the combined influence of both endo- and exopeptidases (56, 69). The combination renders it difficult to discriminate whether peptides differing only by a few amino acids at their terminals were generated by different endopeptidases or by a different extent of exopeptidase activity. One way of overcoming this uncertainty is by visualization through programs like Peptigram. The distinct clustered regions of peptides suggest that peptides within this region are a product of the same endopeptidases but modified by exopeptidases. Visible clusters like these are indeed present in **Figure 5A** and in our **Supplementary Data Sheet 1**.

### Antimicrobial Peptides in Wound Fluids

AMPs have recently received a lot of attention as novel antimicrobials with a potential to substitute antibiotics (70, 71). Their antimicrobial mechanism allows for broad spectrum bactericidal properties (72). AMPs also have comparatively benign environmental and ecological consequences as compared to widely used antiseptics and antibiotics (70, 71, 73). The identification of novel AMPs has been subject to *in vitro* as well as *in silico* experiments. Deep-AmPEP30 is a recently published open source tool in the arsenal of identification of short AMPs and has proven to outperform many state-of-the-art algorithms (11). We used Deep-AmPEP30, in combination with the relative peptide abundance, as a tool to estimate and predict the antimicrobial environment in the different wound types. The reasoning is based on the fact that the antimicrobial effect of an antimicrobial peptide is a function of both its potency, often described as its MIC (Minimum Inhibitory Concentration), and its concentration (72). Interestingly, the antimicrobial score was found to be significantly higher within the infected group than in the non-infected and acute group, as seen in **Figure 6**. However, the antimicrobial prediction score may not always accurately predict the bactericidal or growth inhibiting properties of a peptide (MIC), as peptides may have synergistic effects which are not replicable in a test tube but requires the environment present in a wound to be effective (15, 74–76).

The major protein substrates contributing to AMPs differed between the groups but were mainly fibrinogen alpha chain and hemoglobin subunit alpha and beta chains. Several articles have shown that peptides derived from this region have antimicrobial properties as can be seen in **Table 1**, validating our findings. Interestingly, peptides derived from this region are abundant in infected wounds, which suggests that hemoglobin degradation contributes to body’s defense against microbes in the wound environment. Indeed, Mak et al. showed that peptides such as AHHFGKEFTPPVQAAYQKVVAGVANALAHKYH derived from this hemoglobin region act as AMPs in the female genital tract (14, 15). Furthermore, **Table 1** shows that several other peptides such as MVHLTPEEKSAVTALWGKVNV (36), AHLPAEFTPAVHA (19) and SFPTTKTYFPHFDLSHGSAQVK (14) also have proven antimicrobial properties. These results are compatible with previous findings showing that diverse protein families may give rise to AMPs after proteolysis (77).

### Potential Biomarkers in Wound Fluids

Diagnosing infection in its early stages will significantly decrease the cost of infection-related illnesses (78). The clinical courses of infected and non-infected wounds are widely different and the articles by Cutting (79) and Ligi et al. (80) showed that there are differences in the corresponding wound fluids. Hypothetically, this should translate to differences in the peptidomes, enabling a peptidomic approach to diagnosing wounds by discovering unique peptides or epitopes which can be used as biomarkers. Indeed, much research has been conducted in order to find peptide biomarkers in chronic and acute diseases such as acute pancreatitis (37), Alzheimer’s (48) and different forms of cancer (81, 82). Biomarkers would preferably exist exclusively in the intersection of infected peptidomes and not in any other peptidome, but unfortunately, very few of these peptides exist. Therefore, a more realistic application of peptides as biomarkers relies on differences in concentrations, translating to differences in signal intensity. Alternatively, a binary method could be applied by using a combination of peptides more common in infected wounds. Based on our results, peptides derived from the regions outlined in **Table 1** may serve as interesting biomarker candidates. as they seem most abundant in infected wounds. Notably, peptides from these regions have been validated previously as biomarkers for e.g. inflammatory arthritis (39, 45) and renal carcinoma (40). Interestingly, many of the peptides previously identified as biomarkers for different pathological states (such as cancer, inflammatory gastrointestinal diseases and autoimmune conditions in patient samples of bodily fluids such as serum, urine and synovial fluid) are derived from similar protein regions, suggesting a link between wound infection and other pathological conditions involving inflammation activation. Moreover, peptides from some of these regions, for example the mentioned region in HBB (111–146), and region 111–129 of hemoglobin subunit alpha have been shown to potentiate smooth muscle contractions and bind to LPS (19, 35), adding further relevance to our findings. Instead of identifying unique peptide sequences as biomarkers, one could also target any difference in N and C-terminal sequences using antibodies for the diagnosis of infection. By analyzing the spectral count of specific N and C-terminals we found that some terminals are preferred in the various wound fluids, as shown in **Figure 4**. Comparisons between the groups suggest a higher degree of terminal preference in the infected than in the non-infected and acute wound fluids, as indicated by the steepness of the curve. Furthermore, there are indeed specific sequences found to a greater extent in infected wounds rather than non-infected wounds. The most prevalent being the inherent C-terminal of hemoglobin subunit beta (HKYH), which remained intact in many peptides. The increased preference in infected samples could be due to specific protease activity, and/or specific substrate availability. The nature of the prevalent terminals suggests that at least the latter is true, as many of them are part of hemoglobin’s innate terminals. When grouping amino acids based on their acidity and polarity according to side chain property (24), we found similar differences **(** **Supplementary Figure 5** **),** again showing a higher degree of preference in the infected wounds.

As also shown here, the peptidomes of individuals vary greatly, and therefore, investigating specific differences in the peptidome between groups would benefit from larger sets of data to sift out unreliable findings and intensify any existing differences. It is of note that our dataset was limited and aimed at generating proof of principle data, establish new bioinformatics approaches, and validate the methodologies. Nevertheless, the fact that significant differences still emerged between the different peptidomes, despite the limited patient number, illustrates the power of the present approach in detecting subtle qualitative differences in peptide patterns. Future work should therefore include larger patient groups with well-defined wounds, as well as a refinement of the analysis. However, the fact that our limited analysis still identified large differences between the groups demonstrates the power of our combined peptidomics and bioinformatics approach. The methods and conceptual approaches used in this study, the results and their significance are summarized in **Supplementary Data Sheet 2**.

## Data Availability Statement

The datasets presented in this study can be found in online repositories. The names of the repository/repositories and accession number(s) can be found in the article/**Supplementary Material**.

## Ethics Statement

The studies involving human participants were reviewed and approved by the Ethics Committee at Lund University. The patients/participants provided their written informed consent to participate in this study.

## Author Contributions

EH, KW, AS, and SK participated in the planning, design and interpretation of experiments and results. KS collected the samples, and MP, JP, and JC prepared the samples for MS/MS. SK performed the LC-MS/MS and generated the dataset. EH and KW performed the computational analysis. AS and SK contributed with the methodology for sample collection and LC-MS/MS to the manuscript. EH and KW wrote the manuscript. All authors contributed to the article and approved the submitted version.

## Funding

This work was supported by grants from Alfred Österlund Foundation, Edvard Welanders Stiftelse and Finsenstiftelsen (Hudfonden), Lars Hiertas Memorial Foundation, Åke Wibergs Foundation, LEO Foundation, O.E. and Edla Johanssons Foundation, the Royal Physiographic Society in Lund, Swedish Research Council (project 2017-02341), and the Swedish Government Funds for Clinical Research (ALF).

## Conflict of Interest

The authors declare that the research was conducted in the absence of any commercial or financial relationships that could be construed as a potential conflict of interest.

## Supplementary Material

Supplementary Table 1 The file contains all data from the literature search. Each sheet represents one article and contains the original data extracted from the article alongside every matching sequence in our data as provided by our algorithm.

Supplementary Table 2 Table of general characteristics. The table shows the descriptive characteristics of the different peptidomes as indicated.

Supplementary Table 3 Amino acid distribution of complete sequences. Table showing the amino acids distribution of the complete sequences.

Supplementary Table 4 The excel file contains the unaltered data retrieved from the Proteasix protease prediction tool.

Supplementary Table 5 The excel file containing all the unaltered data retrieved from the antimicrobial peptide prediction tool AmPEP.

Supplementary Data Sheet 1 The folder contains all the peptigrams in their original format.

Supplementary Data Sheet 2 Figure summarizing the bioinformatics approaches, the results, and their significance as presented in the article.

Supplementary Figure 1 Total spectral count in samples. The bar chart shows the spectral count and standard deviation in samples for the acute, non-infected and infected samples.

Supplementary Figure 2 Figure of outliers. **(A)** The figure shows the average spectral count over the occurrence in patients. The figure was used to identify outlying sequences with increased spectral counts. **(B)** The table shows the outlying sequences alongside some characteristics.

Supplementary Figure 3 Protein pie charts without hemoglobin. The figure shows pie charts where hemoglobin derived peptides have been excluded from the dataset. The size of the pies are proportional to the total spectral count of peptide deriving from each protein.

Supplementary Figure 4 Amino acid distribution with aureolysin. The pie charts show the amino acid distribution of complete sequences, P1 and P1’ position of the peptides. The pie charts representing aureolysin degradation were made in the same way but using the dataset from the degradation of thrombin by aureolysin (32).

Supplementary Figure 5 Identification of the most abundant characteristics behind the N- and C-terminals. **(A)** Scatter plots visualizing the distribution of the characteristics of N-terminals (the first 4 amino acids) and C-terminals (the 4 last amino acids) by plotting the aggregated spectral count after grouping amino acids by side chain properties (22). A difference in curvature in the different groups can be seen. **(B)** Table showing the sequence and average spectral count of the most abundant terminals, which are highlighted in the scatter plots.
