# Supplementary material for: Bioinformatic Analysis of the Wound Peptidome Reveals Potential Biomarkers and Antimicrobial Peptides
Source: Front Immunol. 2021 Feb 3;11:620707. doi: 10.3389/fimmu.2020.620707 (PMC7888259; doi:10.3389/fimmu.2020.620707)
Supplement: Supplementary Table 2 — Table of general characteristics. The table shows the descriptive characteristics of the different peptidomes as indicated. [file Table_2.docx]

|  | **Acute Wound Fluid (n=5)** | **Non-infected (n=3)** | **Infected (n=3)** |
| --- | --- | --- | --- |
| **# of unique peptides** | 7175 | 7013 | 6483 |
| **# peptides with spectral count > 3** | 2034 | 1054 | 1419 |
| **Average mass ± SD** | 1403.05 ± 32.892 | 1319.42 ± 86.43 | 1465.91 ± 170.058 |
| **Average length ± SD** | 12.64 ± 0.360 | 12.07 ± 0.87 | 13.47 ± 1.632 |
| **# of proteins** | 232 | 124 | 102 |
