# Supplementary material for: Bioinformatic Analysis of the Wound Peptidome Reveals Potential Biomarkers and Antimicrobial Peptides
Source: Front Immunol. 2021 Feb 3;11:620707. doi: 10.3389/fimmu.2020.620707 (PMC7888259; doi:10.3389/fimmu.2020.620707)
Supplement: Supplementary Data Sheet 1 — The folder contains all the peptigrams in their original format. [file DataSheet_1.zip › Supplementary_2_All_peptigrams/WF/HBA_WF.pdf]

This website uses cookies to ensure you get the best experience on our website.Learn more
Got it!

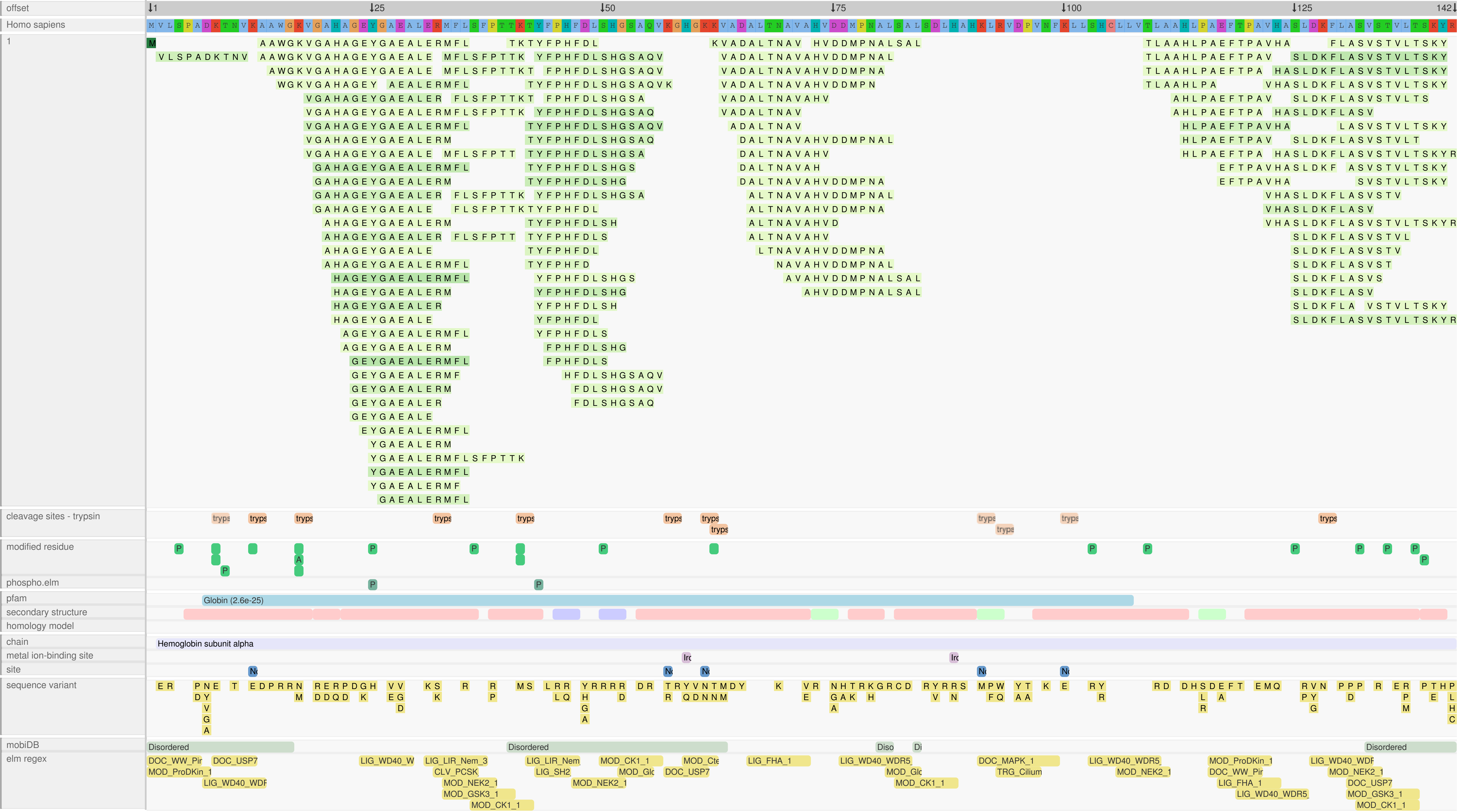

offset
Homo sapiens
1
cleavage sites - trypsin
modified residue
phospho.elm
pfam
secondary structure
homology model
chain
metal ion-binding site
site
sequence variant
mobiDB
elm regex
1
25
50
75
100
125
142
Globin (2.6e-25)
Hemoglobin subunit alpha
Disordered
Disordered
Disordered
DOC_WW_Pin1
DOC_USP7
MOD_ProDKin_1
LIG_WD40_WDR5
LIG_LIR_Nem_3
CLV_PCSK
MOD_NEK2_1
MOD_GSK3_1
MOD_CK1_1
LIG_SH2
LIG_FHA_1
DOC_MAPK_1
TRG_Cilium
