# Supplementary figures and images for: Bioinformatic Analysis of the Wound Peptidome Reveals Potential Biomarkers and Antimicrobial Peptides
Source: Front Immunol. 2021 Feb 3;11:620707. doi: 10.3389/fimmu.2020.620707 (PMC7888259; doi:10.3389/fimmu.2020.620707)

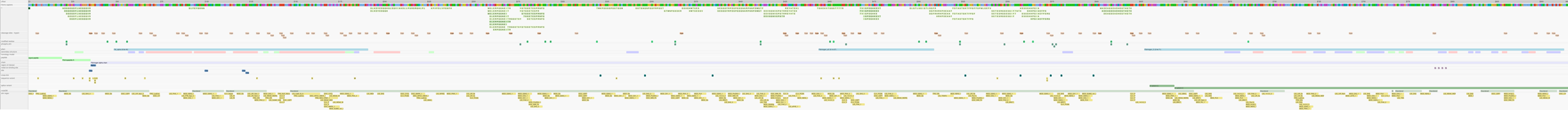

Supplement: Supplementary Data Sheet 1 — The folder contains all the peptigrams in their original format. [file DataSheet_1.zip › Supplementary_2_All_peptigrams/INF/FIBA_INF.pdf]

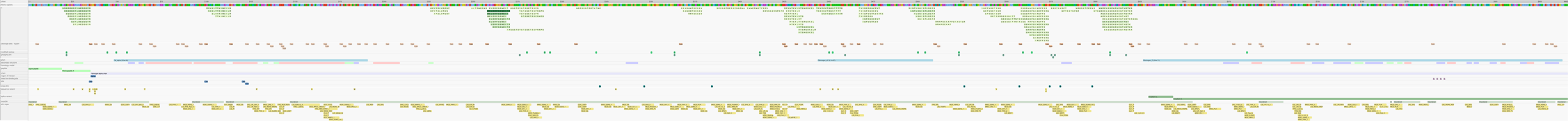

Supplement: Supplementary Data Sheet 1 — The folder contains all the peptigrams in their original format. [file DataSheet_1.zip › Supplementary_2_All_peptigrams/NINF/FIBA_NINF.pdf]

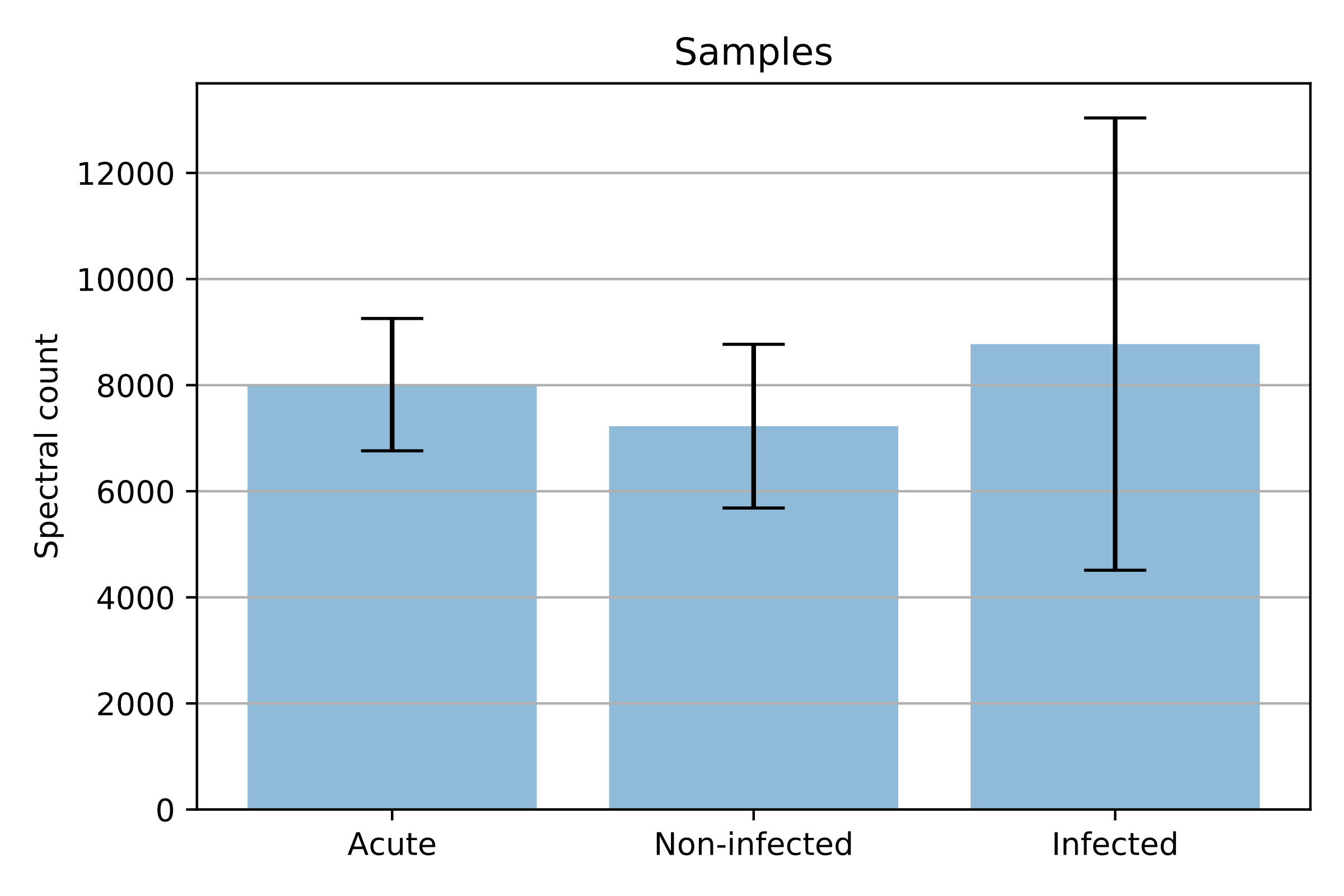

Supplement: Supplementary Figure 1 — Total spectral count in samples. The bar chart shows the spectral count and standard deviation in samples for the acute, non-infected and infected samples. [file Image_1.jpeg]

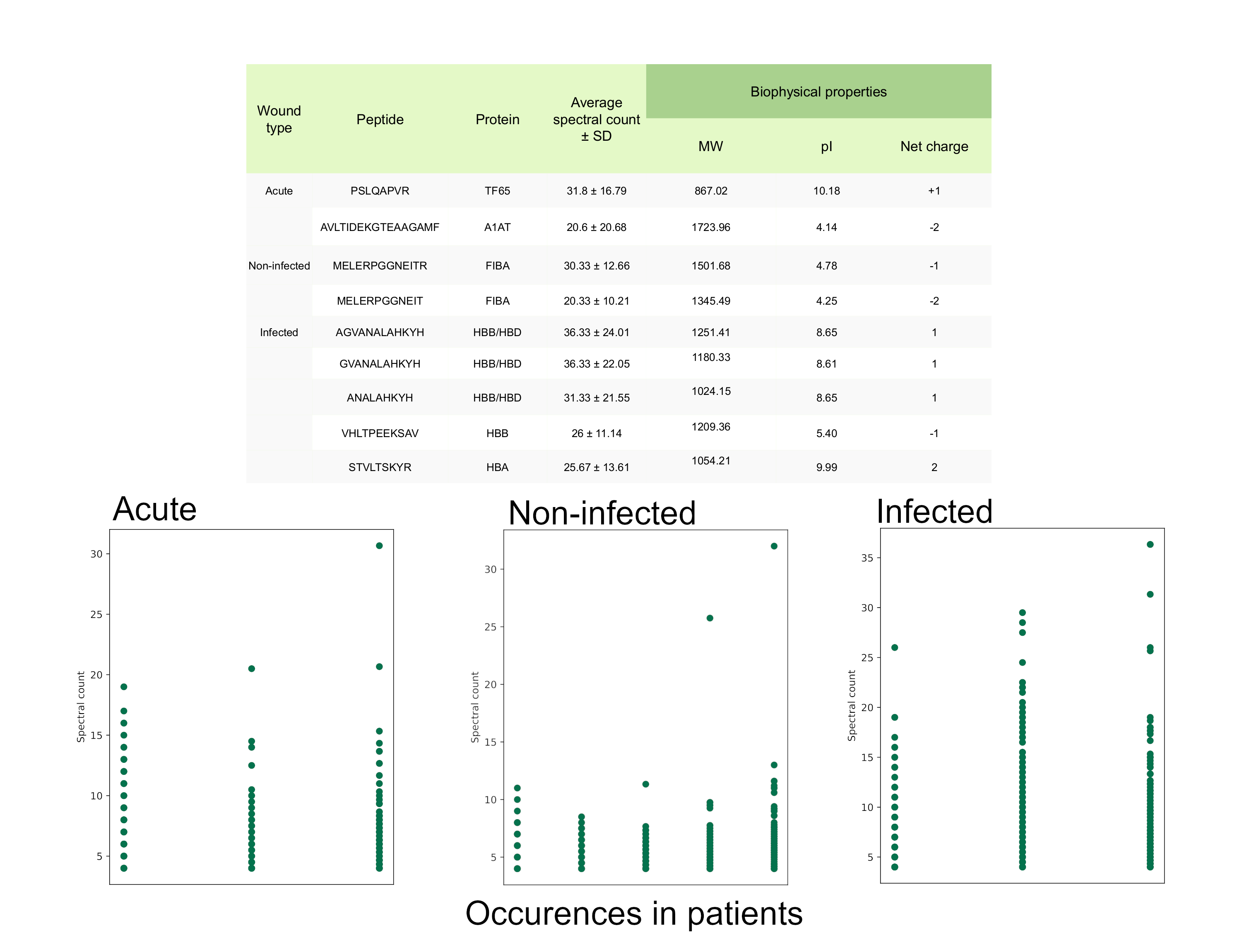

Supplement: Supplementary Figure 2 — Figure of outliers. (A) The figure shows the average spectral count over the occurrence in patients. The figure was used to identify outlying sequences with increased spectral counts. (B) The table shows the outlying sequences alongside some characteristics. [file Image_2.png]

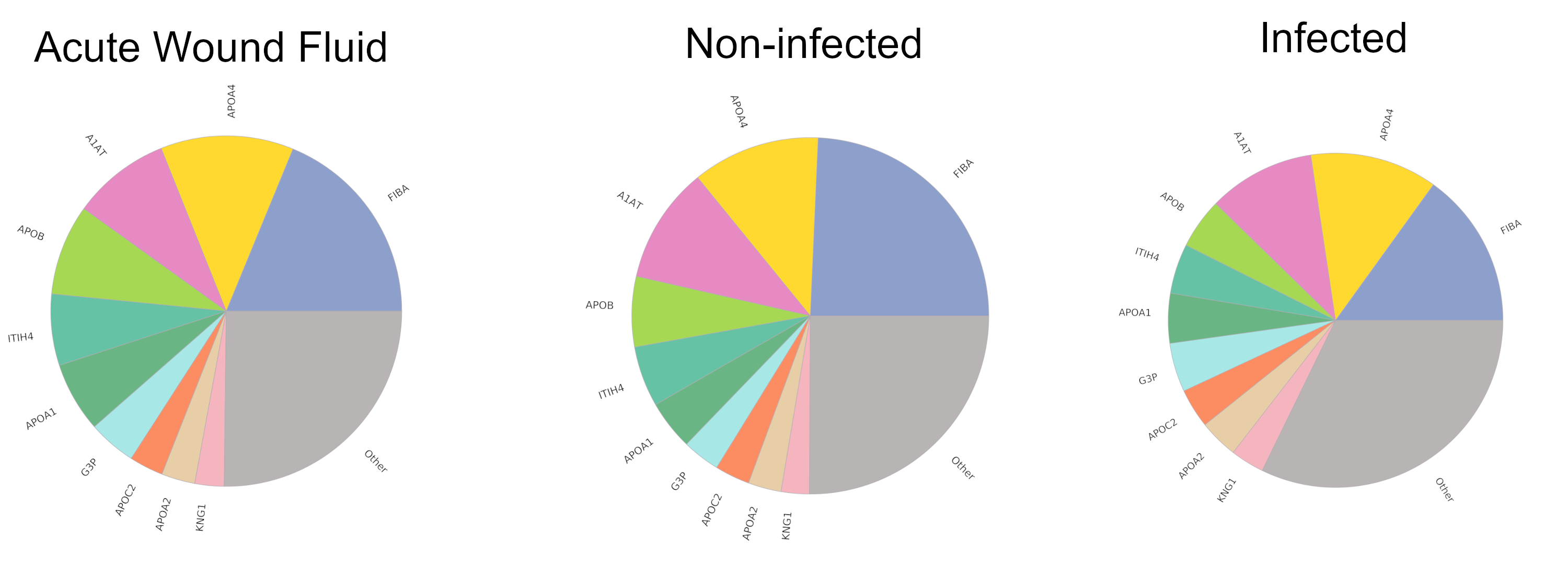

Supplement: Supplementary Figure 3 — Protein pie charts without hemoglobin. The figure shows pie charts where hemoglobin derived peptides have been excluded from the dataset. The size of the pies are proportional to the total spectral count of peptide deriving from each protein. [file Image_3.png]

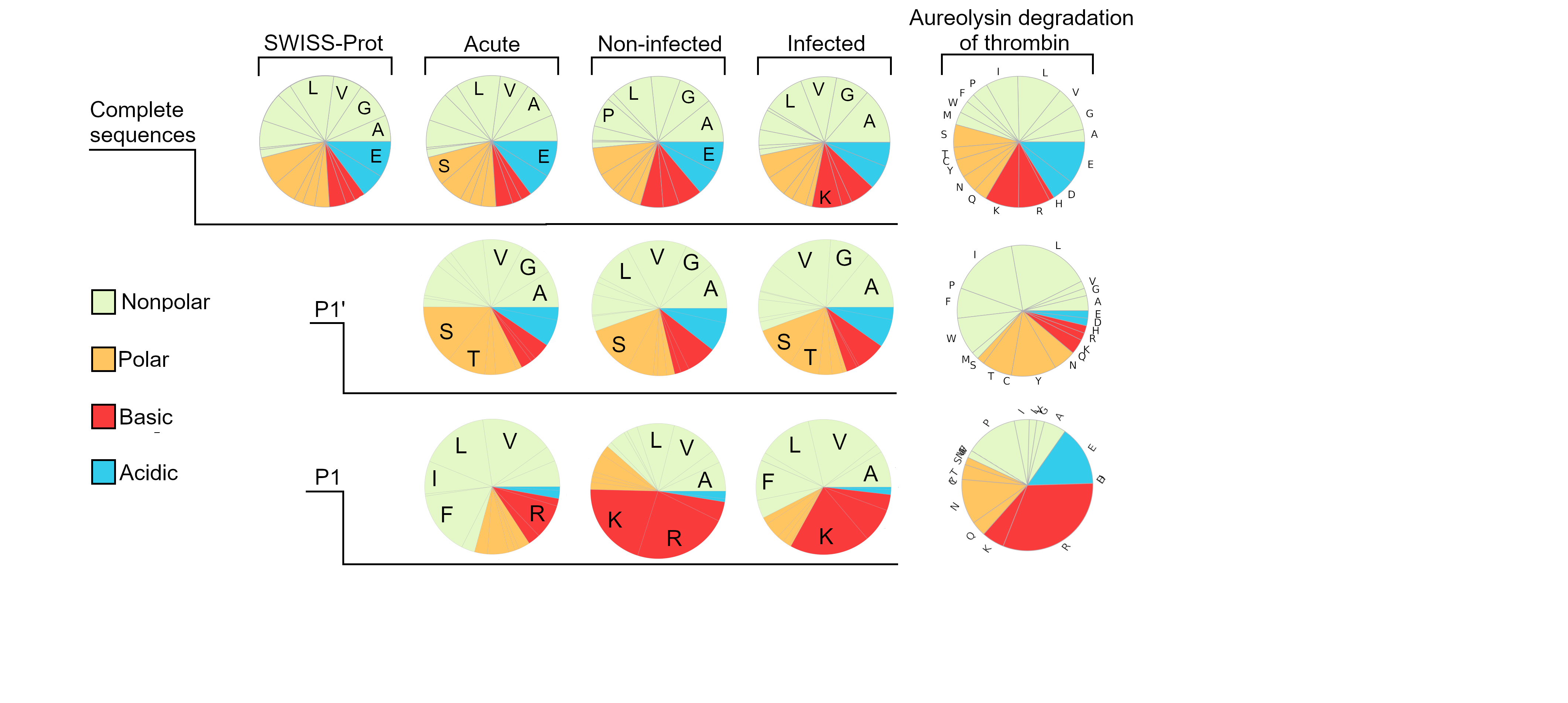

Supplement: Supplementary Figure 4 — Amino acid distribution with aureolysin. The pie charts show the amino acid distribution of complete sequences, P1 and P1’ position of the peptides. The pie charts representing aureolysin degradation were made in the same way but using the dataset from the degradation of thrombin by aureolysin (32). [file Image_4.png]

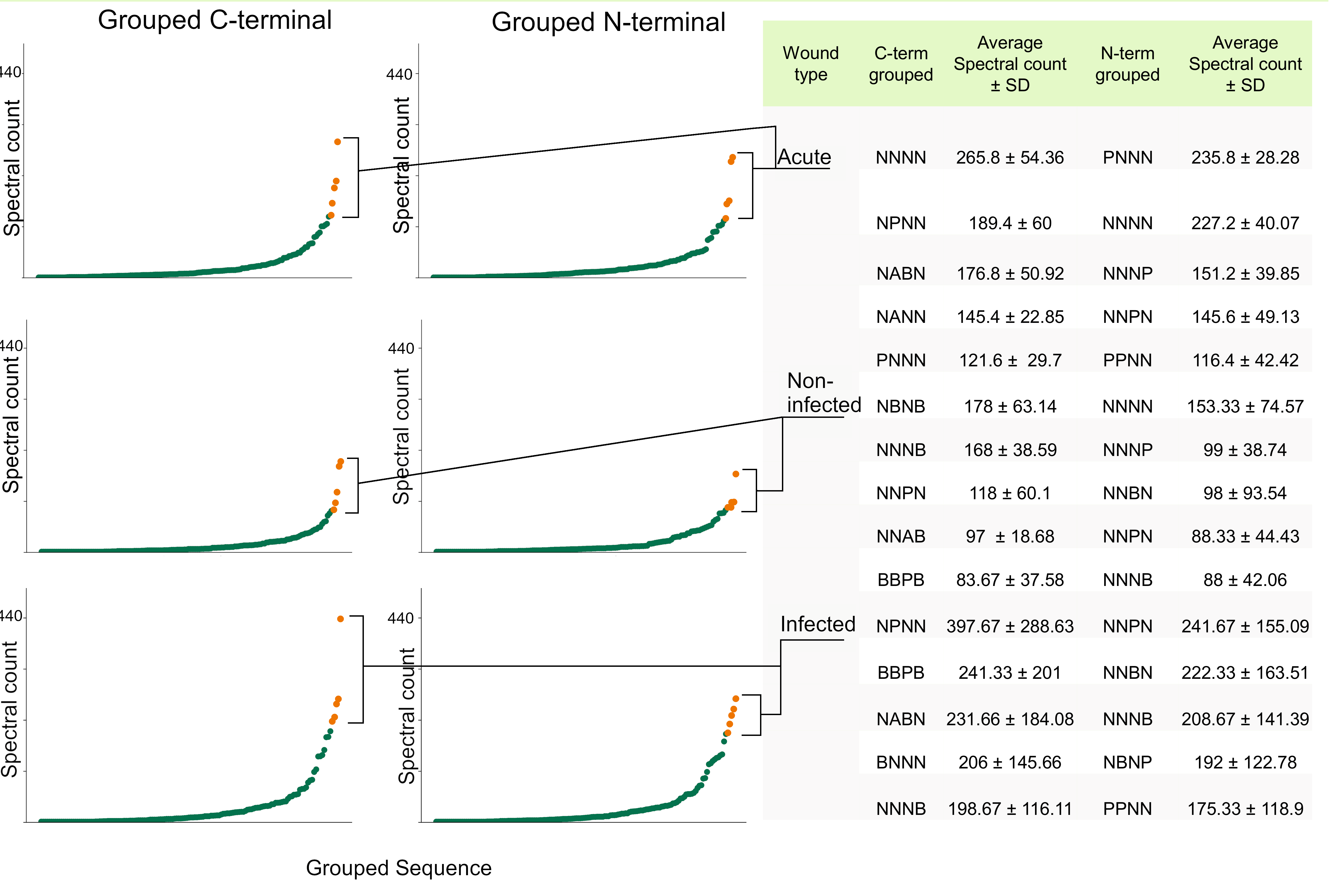

Supplement: Supplementary Figure 5 — Identification of the most abundant characteristics behind the N- and C-terminals. (A) Scatter plots visualizing the distribution of the characteristics of N-terminals (the first 4 amino acids) and C-terminals (the 4 last amino acids) by plotting the aggregated spectral count after grouping amino acids by side chain properties (22). A difference in curvature in the different groups can be seen. (B) Table showing the sequence and average spectral count of the most abundant terminals, which are highlighted in the scatter plots. [file Image_5.png]
