# Supplementary material for: Bioinformatic Analysis of the Wound Peptidome Reveals Potential Biomarkers and Antimicrobial Peptides
Source: Front Immunol. 2021 Feb 3;11:620707. doi: 10.3389/fimmu.2020.620707 (PMC7888259; doi:10.3389/fimmu.2020.620707)
Supplement: Supplementary Data Sheet 2 — Figure summarizing the bioinformatics approaches, the results, and their significance as presented in the article. [file DataSheet_2.pdf]

Methodology

LC-MS/MS

+

Sorting algorithms

+

3rd party applications

+

Literature validation

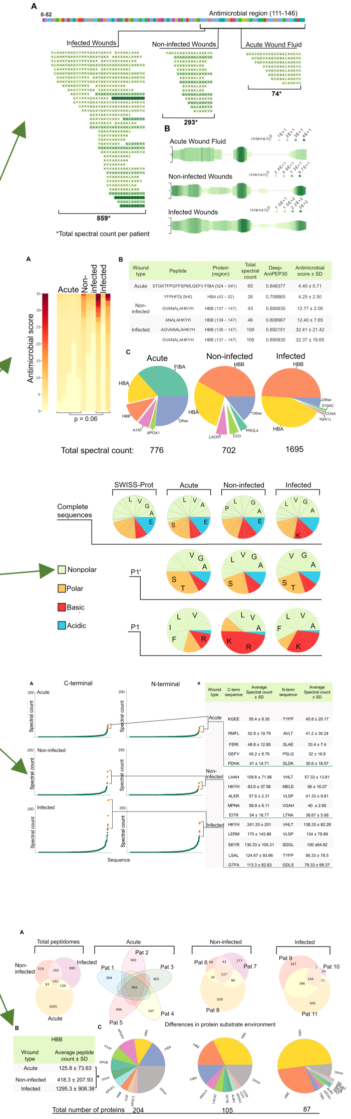

Results	Significance
**Differences in peptide levels,** as exemplified by the abundance of the region 111-147 in hemoglobin subunit beta in infected wounds.	The methodology can be used to define peptides as **biomarkers.** Warrants further studies on hemoglobin-derived peptides as diagnostic markers.
Infected wounds contain **antimicrobial peptides** derived from specific proteins such as region 111-147 in hemoglobin subunit beta.	The methodology can be used for large scale **antimicrobial peptide screening**. The results suggests presence of specific **degradation patterns** in infected wounds.
**N and C-terminals** of peptides differ between the different wound types. Some amino acid terminals are especially abundant.	N and C-terminal epitopes may be used as targets for **antibodies or aptamers, of diagnostic relevance.** The results indicate differences in the **protease environment** in infected wounds.
The peptidome is highly **variable**, but patterns emerge when looking at a **grand scale**. There is a large difference in **protein substrates** between the different wound types.	Large scale bioinformatics analyses are relevant in the future studies of different wounds and the development of **new tools for wound assessment**.
Many peptides found to be abundant in MS/MS-data have been previously described as biomarkers in other disease states.	Validating the methodology and finding correspondence in literature supports obtained data and generalizes the findings.
