# Supplementary material for: Bioinformatic Analysis of the Wound Peptidome Reveals Potential Biomarkers and Antimicrobial Peptides
Source: Front Immunol. 2021 Feb 3;11:620707. doi: 10.3389/fimmu.2020.620707 (PMC7888259; doi:10.3389/fimmu.2020.620707)
Supplement: Supplementary Data Sheet 1 — The folder contains all the peptigrams in their original format. [file HBB_INF.pdf › Supplementary_2_All_peptigrams/INF/HBB_INF.pdf]

This website uses cookies to ensure you get the best experience on our website.Learn more
Got it!

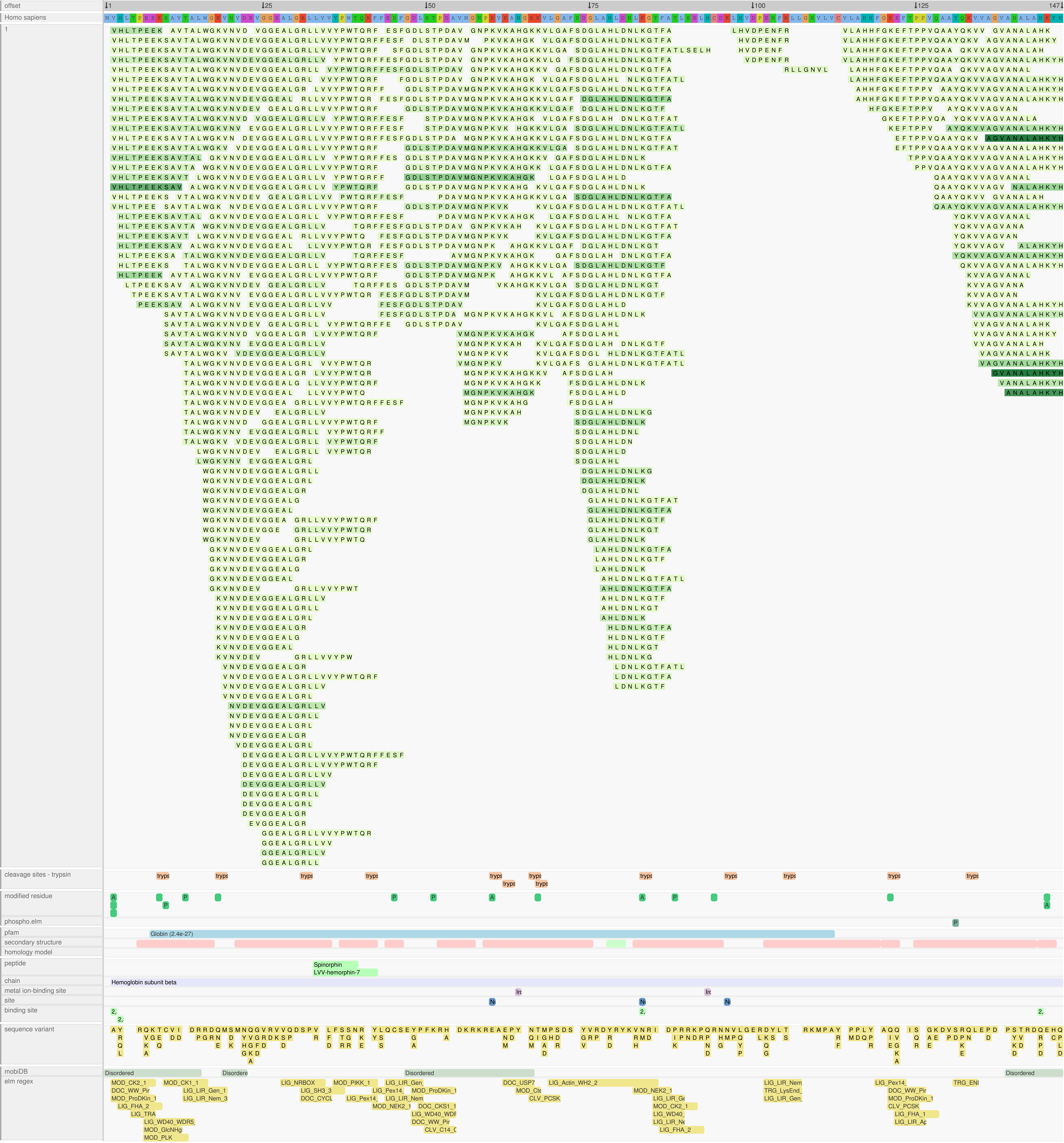
offset
Homo sapiens
1
cleavage sites - trypsin
modified residue
phospho.elm
pfam
Globin (2.4e-27)
secondary structure
homology model
peptide
Spinorphin
LVV-hemorphin-7
chain
Hemoglobin subunit beta
metal ion-binding site
site
binding site
sequence variant
mobiDB
Disordered
elm regex
MOD_CK2_1
MOD_CK1_1
DOC_WW_Pin1
LIG_LIR_Gen_1
MOD_ProDKin_1
LIG_LIR_Nem_3
LIG_FHA_2
LIG_TRAF2_1
LIG_WD40_WDR5
MOD_GlcNHglycan
MOD_PLK
LIG_NRBOX
MOD_PIKK_1
LIG_SH3_3
DOC_CYCLIN
LIG_Pex14
MOD_NEK2_1
DOC_CKS1_1
DOC_USP7
LIG_Actin_WH2_2
CLV_PCSK
CLV_C14_Caspase3-7
TRG_LysEnd
TRG_ENDOCYTIC
