# Supplementary material for: Bioinformatic Analysis of the Wound Peptidome Reveals Potential Biomarkers and Antimicrobial Peptides
Source: Front Immunol. 2021 Feb 3;11:620707. doi: 10.3389/fimmu.2020.620707 (PMC7888259; doi:10.3389/fimmu.2020.620707)
Supplement: Supplementary Data Sheet 1 — The folder contains all the peptigrams in their original format. [file HBB_NINF.pdf › Supplementary_2_All_peptigrams/NINF/HBB_NINF.pdf]

This website uses cookies to ensure you get the best experience on our website.Learn more
Got it!

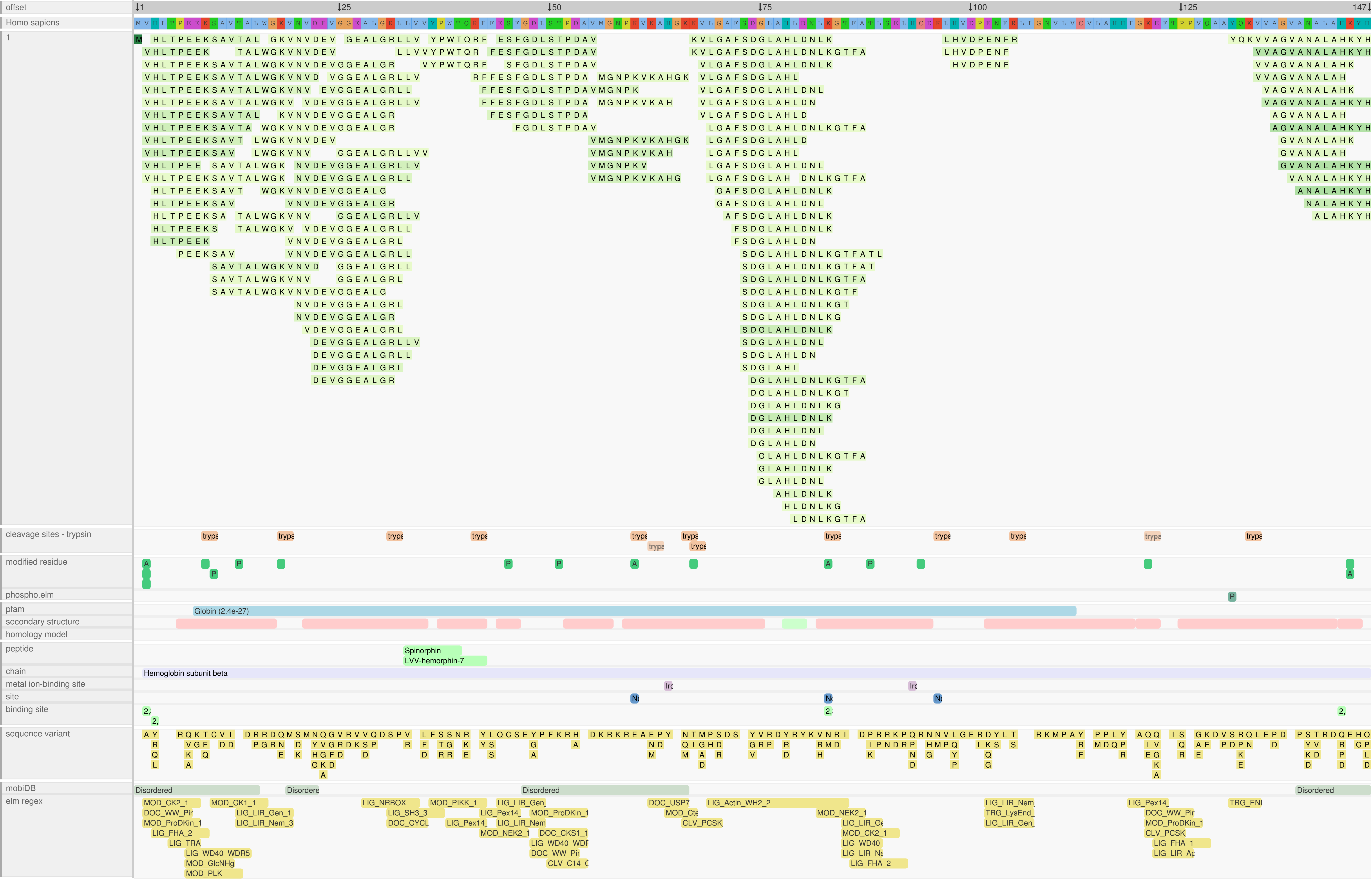

offset
1
25
50
75
100
125
147
Homo sapiens
MVHLTPEEKSAVTALWGKVNVDEVGGEALGRLLVVYPWTQRFFESFGDLSTPDAVMGNPKVKAHGKKVLGAFSDGLAHLDNLKGTFATLSELHCDKLHVDPENFRLLGNVLVCVLAHHFGKEFTPPVQAAYQKVVAGVANALAHKYH
cleavage sites - trypsin
modified residue
phospho.elm
pfam
Globin (2.4e-27)
secondary structure
homology model
peptide
Spinorphin
LVV-hemorphin-7
chain
Hemoglobin subunit beta
metal ion-binding site
site
binding site
sequence variant
mobiDB
Disordered
elm regex
