# Supplementary material for: Bioinformatic Analysis of the Wound Peptidome Reveals Potential Biomarkers and Antimicrobial Peptides
Source: Front Immunol. 2021 Feb 3;11:620707. doi: 10.3389/fimmu.2020.620707 (PMC7888259; doi:10.3389/fimmu.2020.620707)
Supplement: Supplementary Data Sheet 1 — The folder contains all the peptigrams in their original format. [file HBB_WF.pdf › Supplementary_2_All_peptigrams/WF/HBB_WF.pdf]

This website uses cookies to ensure you get the best experience on our website.Learn more
Got it!

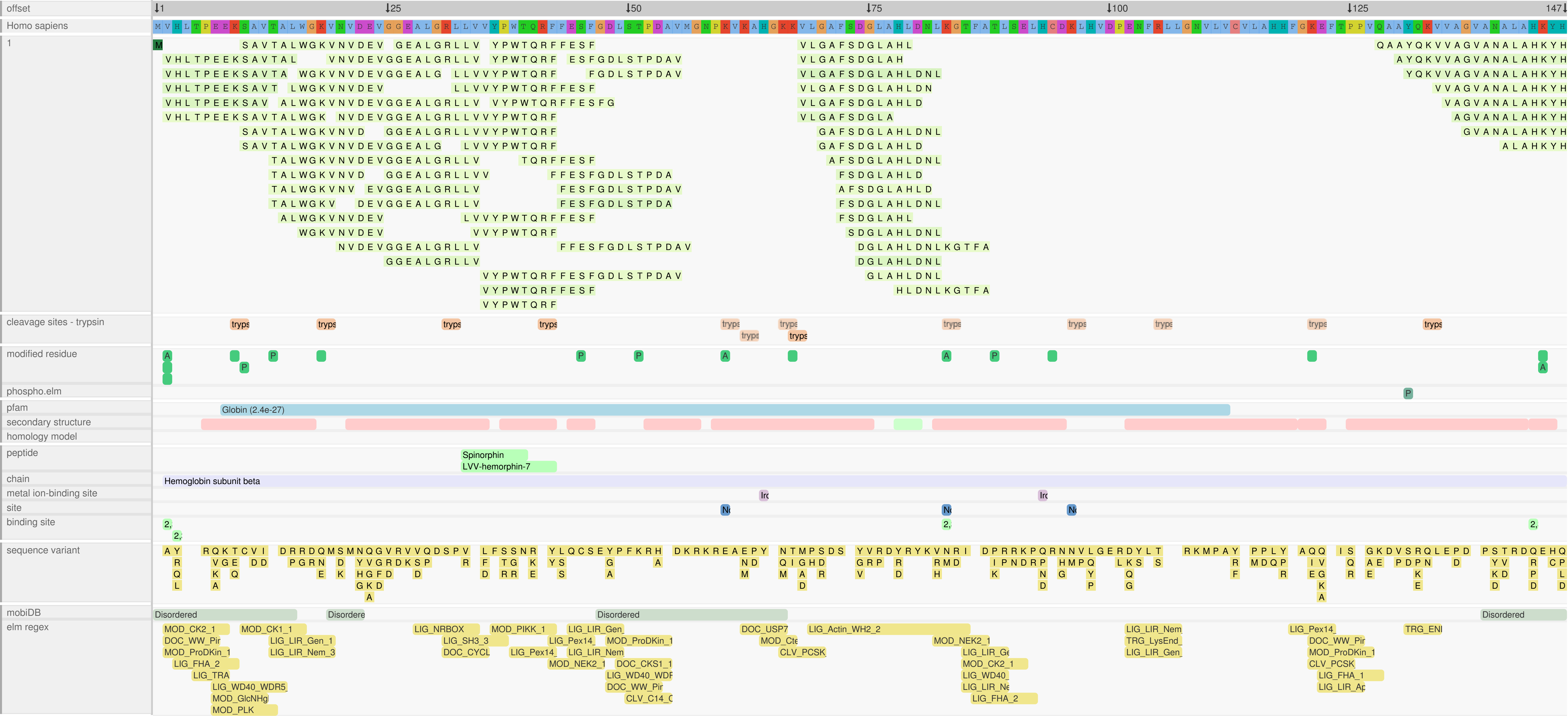

offset
1
25
50
75
100
125
147
Homo sapiens
M V H L T P E E K S A V T A L W G K V N V D E V G G E A L G R L L V V Y P W T Q R F F E S F G D L S T P D A V M G N P K V K A H G K K V L G A F S D G L A H L D N L K G T F A T L S E L H C D K L H V D P E N F R L L G N V L V C V L A H H F G K E F T P P V Q A A Y Q K V V A G V A N A L A H K Y H
cleavage sites - trypsin
modified residue
phospho.elm
pfam
Globin (2.4e-27)
secondary structure
homology model
peptide
Spinorphin
LVV-hemorphin-7
chain
Hemoglobin subunit beta
metal ion-binding site
site
binding site
sequence variant
mobiDB
Disordered
elm regex
MOD_CK2_1
DOC_WW_Pin
MOD_ProDKin_1
LIG_FHA_2
MOD_CK1_1
LIG_LIR_Gen_1
LIG_LIR_Nem_3
LIG_NRBOX
LIG_SH3_3
MOD_PIKK_1
LIG_Pex14
MOD_NEK2_1
DOC_CKS1_1
LIG_Actin_WH2_2
MOD_NEK2_1
