# Supplementary material for: Bioinformatic Analysis of the Wound Peptidome Reveals Potential Biomarkers and Antimicrobial Peptides
Source: Front Immunol. 2021 Feb 3;11:620707. doi: 10.3389/fimmu.2020.620707 (PMC7888259; doi:10.3389/fimmu.2020.620707)

This website uses cookies to ensure you get the best experience on our website.Learn more
Got it!

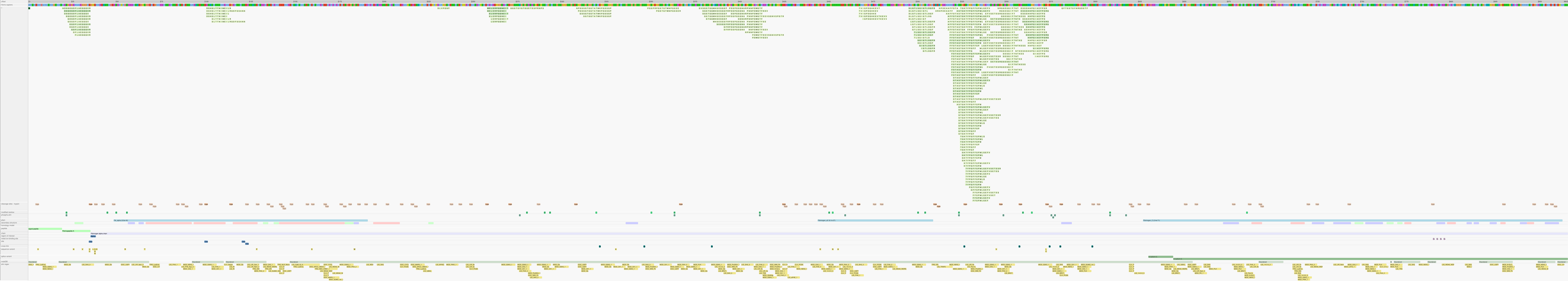

Supplement: Supplementary Data Sheet 1 — The folder contains all the peptigrams in their original format. [file FIBA_WF.pdf › Supplementary_2_All_peptigrams/WF/FIBA_WF.pdf]
